# Tissue-Free vs Tumor-Informed ctDNA Assays for Molecular Residual Disease Detection in Early Triple Negative Breast Cancer

**DOI:** 10.1001/jamaoncol.2026.2833

**Published:** 2026-08-13

**Authors:** Niamh Cunningham, Rosalind J. Cutts, Claire Swift, Maria Coakley, Prithika Sritharan, Kathryn Dunne, Monisha Dewan, Lucy Kilburn, Katie Goddard, Peter Hall, Catherine Harper-Wynne, Iain R. Macpherson, Alicia Okines, Andrew Wardley, Simon Waters, Carlo Palmieri, Matthew Winter, Judith Bliss, Isaac Garcia-Murillas, Nicholas C. Turner

**Affiliations:** 1 The Ralph Lauren Centre for Breast Cancer Research, Royal Marsden Hospital, London, UK; 2 Breast Cancer Now Toby Robins Research Centre, The Institute of Cancer Research, London, UK; 3 Clinical Trials and Statistics Unit, The Institute of Cancer Research, London, UK; 4 Institute of Genetics and Cancer, University of Edinburgh, Edinburgh, UK; 5 Maidstone Hospital, Maidstone and Tunbridge Wells National Health Service (NHS) Trust, Maidstone, UK; 6 School of Cancer Sciences, University of Glasgow, Glasgow, UK; 7 Breast Unit, Royal Marsden Hospital, London, UK; 8 Outreach Research & Innovation Group Ltd, Manchester, UK; 9 Velindre Cancer Centre, Velindre University NHS Trust, Cardiff, UK; 10 The Clatterbridge Cancer Centre NHS Foundation Trust, Liverpool, UK; 11 Weston Park Cancer Centre, Sheffield Teaching Hospitals NHS Foundation Trust, Sheffield, UK

## Abstract

**Importance:**

Molecular residual disease (MRD) detection has potential to transform the selection of adjuvant therapy. Most evidence is from tumor-informed assays. Tissue-free assays not requiring sequencing of the primary tumor may simplify workflows if they offer accuracy similar to that of tumor-informed assays.

**Objective:**

To evaluate tissue-free circulating tumor DNA (ctDNA) analysis in patients with triple negative breast cancer (TNBC) and compare the tissue-free assay with tumor-informed assay results.

**Design, Setting, and Participants:**

This was a prognostic and exploratory analysis of patients with TNBC at moderate to high risk of recurrence who were participating in the ctDNA surveillance period of c-TRAK TN, a multicenter phase 2 clinical study. Plasma samples were collected from participants every 3 months for up to 2 years after the completion of adjuvant therapy and analyzed prospectively with digital polymerase chain reaction (dPCR). Data for the analysis were from a database lock on September 28, 2021, with subsequent follow-up through January 18, 2023. Data were analyzed from July 2025 to May 2026.

**Intervention or Exposure:**

Tissue-free assay leverages cancer differential methylation patterns to detect ctDNA.

**Main Outcome and Measures:**

Recurrence-free survival by tissue-free ctDNA detection status. Comparison between tissue-free ctDNA detection and both dPCR and whole-exome sequencing−powered multivariant tumor-informed assays.

**Results:**

The analysis included 1026 plasma samples from 159 patients (mean [SD; range] age, 51.4 [11.4; 25.0-78.0] years; 159 females [100%]). The tissue-free assay detected ctDNA in 54 patients (34.0%), with detection strongly associated with risk of recurrence (HR, 27.2; 95% CI, 13.7-54.2; *P* < .001). Among patients with ctDNA detected by both the tissue-free assay and dPCR (42 patients [26%]), tissue-free detection occurred at an earlier time point in 14 patients (33.3%); dPCR detection occurred before tissue-free detection in no patients. Median lead time to recurrence was 7.9 (95% CI, 6.1-10.5) months for tissue-free vs 5.8 (95% CI, 3.3-10.0) months for dPCR (HR, 0.57; 95% CI, 0.34-0.95; *P* = .03). Concordance between tissue-free and multivariant tumor-informed assays was good. Among patients detected by both assays (41 of 133), 12 of 41 (29.3%) had ctDNA detected earlier by multivariant tumor-informed assay with 1 (2.4%) earlier by the tissue-free assay. Median lead times to recurrence were 7.6 (95% CI, 4.6-10.5) months with tissue-free and 7.1 (95% CI, 5.7-10.0) months with multivariant tumor-informed assay (HR, 1.46; 95% CI, 0.87-2.44; *P* = .15).

**Conclusion and Relevance:**

In this prognostic study, tissue-free ctDNA detection during surveillance was strongly prognostic for recurrence in patients with TNBC. Comparable lead times between the tissue-free and multivariant tumor-informed assays support tissue-free MRD detection in a clinical trial setting, permitting ctDNA testing when tissue is not available.

## Key Points

**Question:**

Can tissue-free circulating tumor DNA (ctDNA) detection accurately estimate recurrence in early triple negative breast cancer and how does this compare to tumor-informed approaches?

**Findings:**

This prognostic study of 159 patients treated for triple negative breast cancer found that tissue-free ctDNA detection during molecular residual disease surveillance was highly prognostic for recurrence. The tissue-free assay detected ctDNA with similar lead time to recurrence compared to the multivariant tumor-informed assay and with a longer lead time than digital polymerase chain reaction.

**Meaning:**

These findings support tissue-free ctDNA detection in the molecular residual disease setting, allowing testing when archival tissue is unavailable.

## Teaser

This prognostic study compares tissue-free circulating tumor DNA analysis to tissue-free assay with tumor-informed assay results for estimating recurrence in patients with triple negative breast cancer.

## Introduction

Detection of circulating tumor DNA (ctDNA) has emerged as a potential biomarker to accurately identify patients with early breast cancer (EBC) at higher risk of recurrence. The detection of ctDNA after definitive treatment, termed molecular residual disease (MRD), is strongly prognostic of recurrence,^1,2,3^ preceding clinical relapse by up to 15 months, providing a potential window for therapeutic intervention.^1,2,3,4^

Highly sensitive assays are required for the accurate detection of MRD, where ctDNA may be present at tumor fraction of less than 0.01% (fraction of tumor-derived cell free DNA [cfDNA]) in plasma. High sensitivity has been achieved using tumor-informed assays, in which the patient’s tumor is sequenced to identify patient-specific variants, paired with bespoke assays to track those variants in plasma DNA.^1,5,6^ However, tumor-informed approaches have limitations: tumor tissue may be unavailable for sequencing; the assay design process may fail; the process takes time, with high costs associated with tumor sequencing and panel design; and the potential logistics of delivering patient-specific assays to large numbers of patients in follow-up after cancer treatment may prove challenging.

A highly sensitive ctDNA assay that does not require tissue sequencing—a tissue-free assay—could overcome logistical challenges and simplify widespread adoption. Yet historically, developing such assays has been challenging; approaches targeting known driver variants lacked sufficient sensitivity,^7^ and the phenomenon of clonal hematopoiesis has limited the ability to identify ctDNA based on detection of nondriver variants.^8,9,10^ Advancements in sequencing technology for epigenomic analysis have enabled the development of tissue-free assays that detect DNA methylation patterns unique to ctDNA in plasma.^11,12,13^ These tissue-free approaches have shown substantial potential for MRD detection,^14,15^ although comparisons between tissue-free and tumor-informed assays in paired samples are lacking.

A Trial Using ctDNA Blood Tests to Detect Cancer Cells After Standard Treatment to Trigger Additional Treatment in Early Stage Triple Negative Breast Cancer Patients (cTRAK)^16^ was, to our knowledge, the first prospective ctDNA surveillance study in early triple negative breast cancer (TNBC) to assess whether ctDNA-guided therapy could improve patient outcomes. ctDNA monitoring was conducted with digital polymerase chain reaction (dPCR) assays tracking 1 or 2 patient-specific variants. A subsequent retrospective analysis was performed using a highly sensitive multivariant whole-exome sequencing (WES)−based tumor-informed assay that detected MRD with longer lead time to relapse than dPCR.^17,18^ The objective of this study was to evaluate tissue-free ctDNA analysis in patients with TNBC and compare the tissue-free assay with tumor-informed assay results.

## Methods

This study was sponsored by the UK Institute of Cancer Research (NCT03145961) and approved by its research ethics committee (No. 17/SC/0900). Patients provided written informed consent. The study was conducted in accordance with the Declaration of Helsinki and the Guideline for Good Clinical Practice.

### Patients and Samples

The methods and results of the cTRAK trial have been previously described.^16^ Patients with TNBC at moderate to high risk of relapse were eligible for ctDNA surveillance after adjuvant treatment. Archival tumor tissue and baseline blood samples were received at registration, with surveillance blood samples collected every 3 months for up to 2 years. If and when ctDNA detection by dPCR occurred, eligible patients received pembrolizumab. At each time point, 40 mL of blood was collected in Streck tubes (Streck Corp), sent to a central laboratory, centrifuged on arrival and stored at −70 °C. Imaging assessments are described in the eMethods in Supplement 1.

### Assay Analysis

Tissue-free analysis for ctDNA was performed retrospectively using the Guardant Reveal assay on the Infinity targeted next-generation sequencing (NGS) platform (Guardant Health). Prior analytical validation reported a limit of detection 95% (LOD_95_) of 0.02% with 3- to 4-ng cfDNA input and 0.005% with 15-ng input at 100% analytical specificity.^19^ Additional details are provided in the eMethods in Supplement 1.

Regarding the WES-based tumor-informed multivariant assay, the Residual Disease and Recurrence assay workflow has been previously described (RaDaR, NeoGenomics)^17,18^ and is summarized in the eMethods in Supplement 1. With regard to dPCR design and analysis, patient-personalized dPCR assays tracking 1 to 2 variants were used for prospective ctDNA analysis in cTRAK as previously published^3,16^ and are summarized eMethods in Supplement 1.

### Statistical Analysis

Data for the analysis were collected from the cTRAK trial database on September 28, 2021, along with follow-up data updated through January 18, 2023. The primary objective was to assess the prognostic significance of tissue-free ctDNA detection. Statistical analysis is described in the eMethods in Supplement 1. Data analysis was conducted from July 2025 to May 2026 using R Studio, version 4.2.2 (R Foundation for Statistical Computing). Tests were 2-tailed and *P* < .05 was considered significant.

## Results

Of 208 patients registered in the cTRAK database, 159 patients (mean [SD; range] age, 51.4 [11.4; 25.0-78.0] years; 159 females [100%]) had available samples that were analyzed with the tissue-free assay (eFigure 1 in Supplement 1). Patient characteristics were reflective of TNBC with moderate to high risk of recurrence (eTable 1 in Supplement 1), and median (IQR) follow-up from the start of ctDNA surveillance was 33.9 (18.4-40.9) months. We successfully analyzed 98.6% of plasma samples (1012 of 1026) from 159 patients using the tissue-free assay with cfDNA extracted from a median (range) plasma volume of 3.5 (1.5-4.5) mL. Of the failed samples, 92.9% (13 of 14) were associated with low cfDNA yield.

ctDNA was detected in 12.2% (123 of 1012) samples at median methylation tumor fraction of 0.226% (range: 0.002%-51.039%). Association between tumor fraction, clinical risk, recurrence, and lead times are shown in eFigures 2 to 4 in Supplement 1. At the patient level, ctDNA was detected in at least 1 time point from 34.0% (54 of 159) of patients. At 12 and 24 months, the estimated proportions of patients with ctDNA detected were 29.4% (95% CI, 21.7%-36.3%) and 36.4% (95% CI, 28.0%-43.8%), respectively. In patients who had ctDNA detected before clinical recurrence, the median interval from detection to recurrence was 7.9 (95% CI, 6.1-10.5) months.

**Figure 1.**
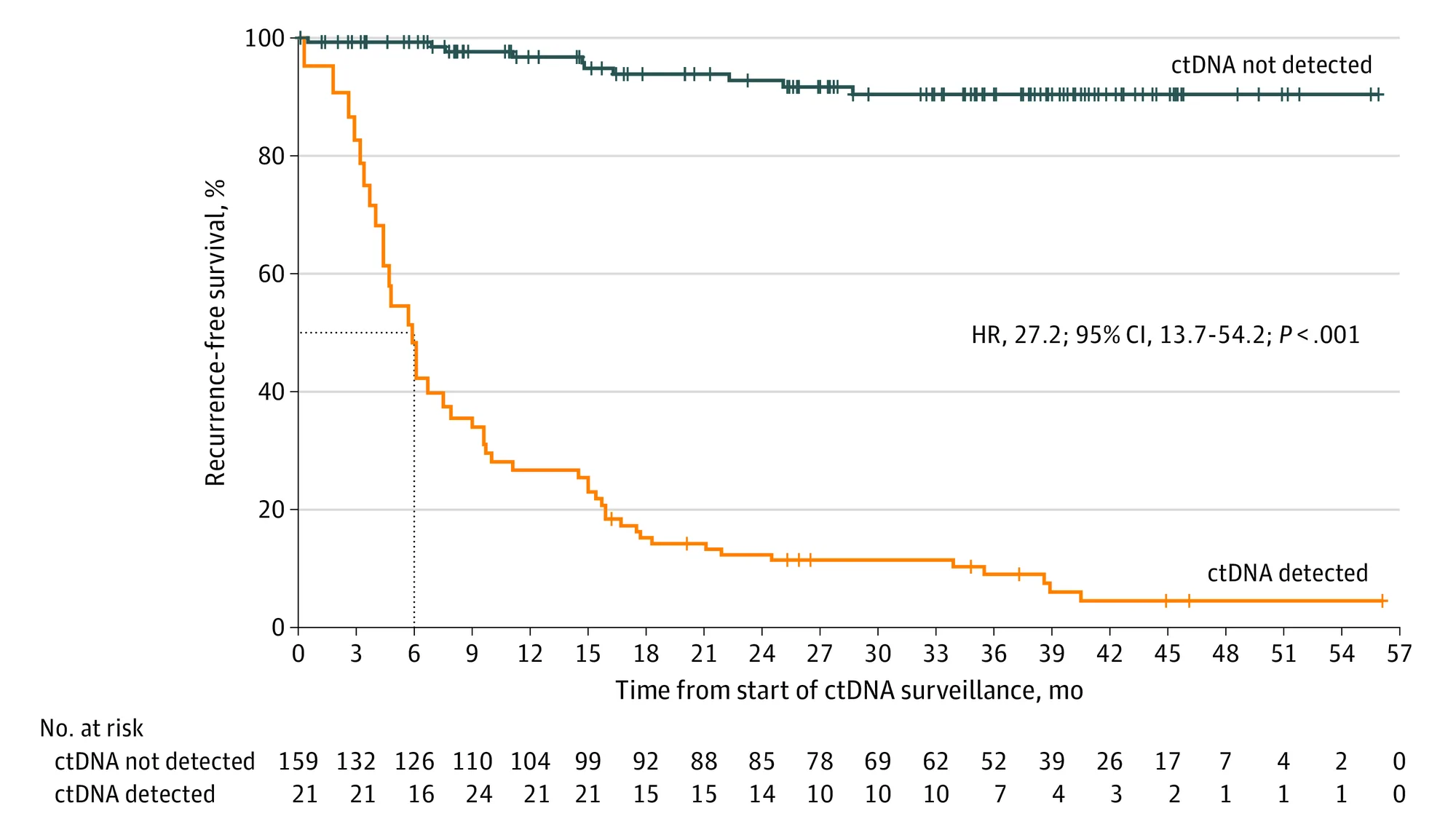
Kaplan-Meier Curve of Circulating Tumor DNA (ctDNA) Detection Results With Tissue-Free Assay During Surveillance for Triple Negative Breast Cancer Recurrence-free survival by ctDNA detection status during molecular residual disease monitoring. Median follow-up was 33.9 months; dotted line shows median recurrence-free survival. HR indicates hazard ratio.

Detection of ctDNA during serial testing with the tissue-free assay was strongly prognostic of recurrence (HR, 27.2; 95% CI, 13.7-54.2; *P* < .001; time dependent Cox model; Figure 1). Among patients who had ctDNA detected at any 1 time point, the median recurrence-free survival from the start of surveillance was 5.9 (95% CI, 4.4-9.7) months and not reached in patients without ctDNA detected. All 3 patients who had ctDNA detected before adjuvant capecitabine experienced relapse, and similarly, all 3 patients who had ctDNA detected during adjuvant noncapecitabine chemotherapy experienced relapse (eFigure 5 in Supplement 1).

### Tissue-Free Assay Accuracy for Identifying Recurrence by 24 Months

To evaluate the diagnostic performance of the tissue-free assay, we assessed the sensitivity to identify recurrence by 24 months of starting surveillance. This analysis included 127 patients (79.9%) with 32 patients censored or lost to follow-up before 24 months and being recurrence free at last assessment. By 24 months, the sensitivity was 84.4% with ctDNA detected in 38 of 45 patients who experienced relapse. The specificity and positive and negative predictive values were 87.8%, 79.2% and 91.1%, respectively. In patients who experienced a distant recurrence by 24 months, the sensitivity was 85.7% with ctDNA detected in 24 of 28 patients. We also evaluated the performance of the test to identify any recurrence (eFigure 6 in Supplement 1).

### Cross Assay Comparison of Tissue-Free and dPCR Assays

**Figure 2.**
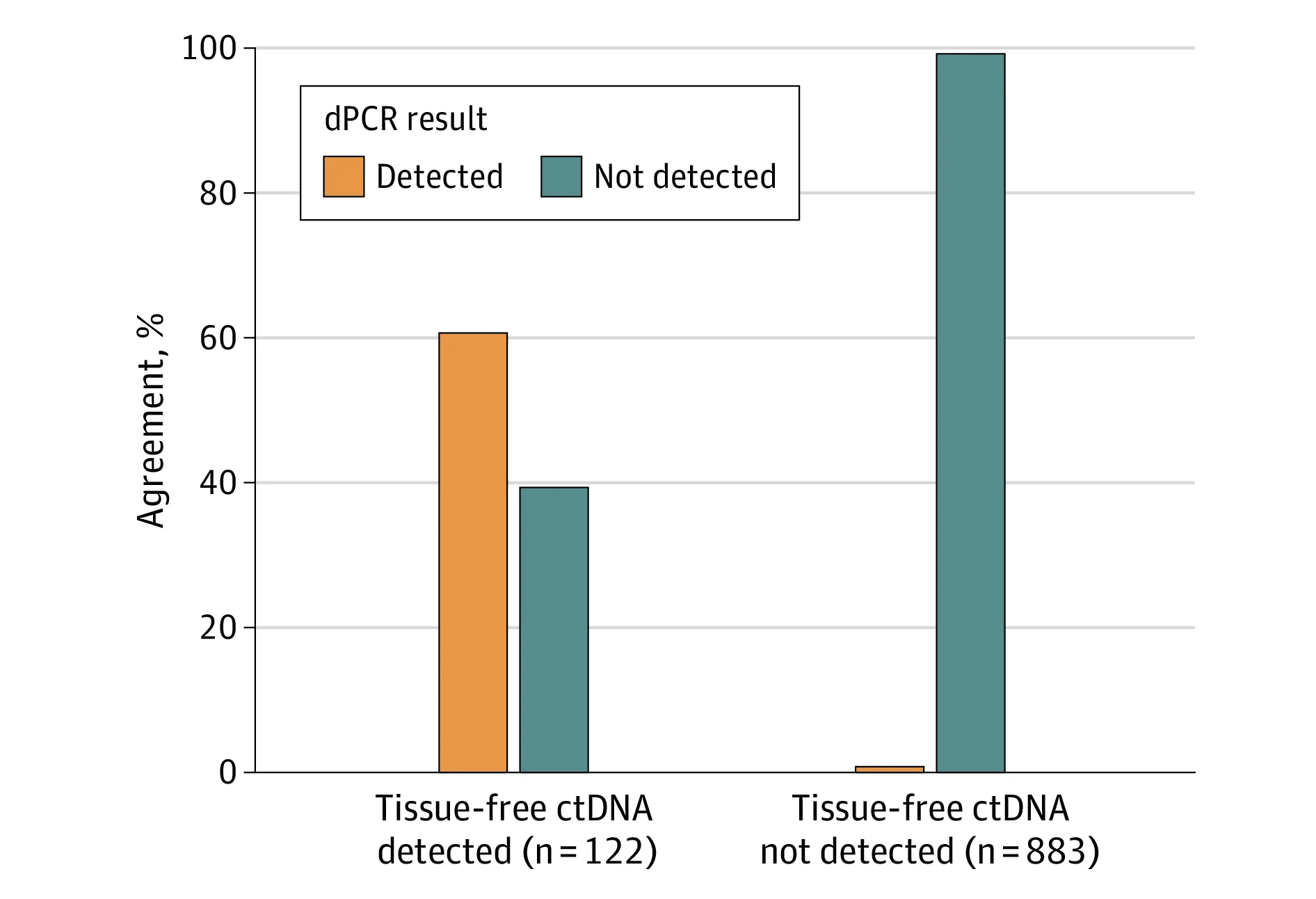
Bar Graph of Agreement Between of Tissue-Free and Digital Polymerase Chain Reaction (dPCR) Detection Results for Circulating Tumor DNA (ctDNA) Of the total samples (n = 1005), 74 results were concordant positive and 876 were concordant negative, with an overall test agreement of 94.5% (95% CI, 92.9%-95.9%).

A total of 159 patients had results from both tissue-free and dPCR assays for comparison in 1005 time points (per patient median [range], 7 [1-11]). Concordant results were observed in 950 of 1005 time points, yielding an overall agreement rate of 94.5% (95% CI, 92.9%-95.9%; Cohen κ, 0.70; 95% CI, 0.63-0.77; Figure 2).

**Figure 3.**
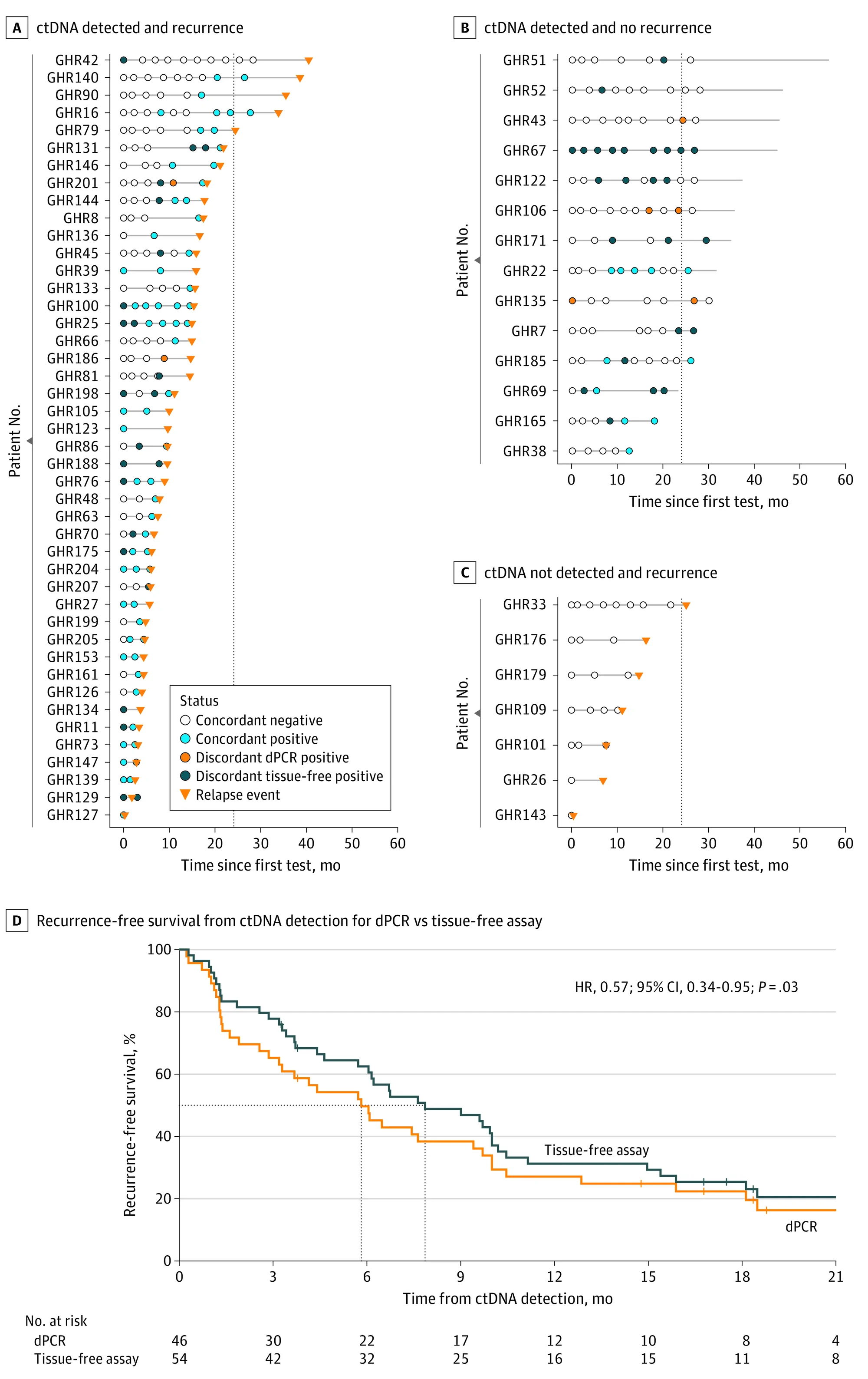
Swimmer Plots and Kaplan-Meier Curve Comparing Tissue-Free to Digital Polymerase Chain Reaction (dPCR) for Detection of Circulating Tumor DNA (ctDNA) A-C, Swimmer plots showing longitudinal data from patients with time points analyzed by both assays. Not shown are 94 patients who did not have ctDNA detected and did not experience recurrence during follow-up. Plots are split by ctDNA detection and recurrence status. D, Kaplan-Meier curve of recurrence-free survival from ctDNA detection for dPCR vs tissue-free assay. The dotted line indicates median recurrence-free survival from ctDNA detection. GHRs are the anonymized patient identifiers. HR indicates hazard ratio.

Overall agreement at the patient level was 143 of 159 patients (89.9%; 95% CI, 84.2%-94.2%). ctDNA was detected by both assays in 42 (26.4%) patients while 101 (63.5%) did not have ctDNA detected by either assay and 16 (10.1%) had discordant results (Figure 3A-C). Among those with ctDNA detected by both assays, initial detection with both assays occurred at the same time point in 28 of 42 patients (66.7%) ; 14 (33.3%) were detected earlier by the tissue-free assay. There were no cases for which dPCR detected ctDNA earlier than the tissue-free assay.

In patients with ctDNA detected, the median lead time from detection to recurrence was 7.9 months (95% CI, 6.1-10.5) months for the tissue-free assay and 5.8 (95% CI, 3.3-10.0) months for dPCR (HR, 0.57; 95% CI, 0.34-0.95; *P* = .03, mixed-effects model; Figure 3D).

### Cross Assay Comparison of Tissue-Free and Multivariant Tumor-Informed ctDNA Assays

Of the 159 patients with samples analyzed by the tissue-free assay, 26 (16.4%) did not have multivariant tumor-informed analysis. The most common reason was lack of WES on archival tumor specimen (21 of 26 [80.8%]) due to insufficient tumor content, DNA or variants identified, or sample contamination (eFigure 1 in Supplement 1). There was no difference observed between the mean volume of plasma sent for analysis by the tissue-free approach and multivariant tumor-informed assay (3.89 vs 3.88 mL; Wilcoxon signed rank test, *P* = .21; eFigure 7 in Supplement 1).

**Figure 4.**
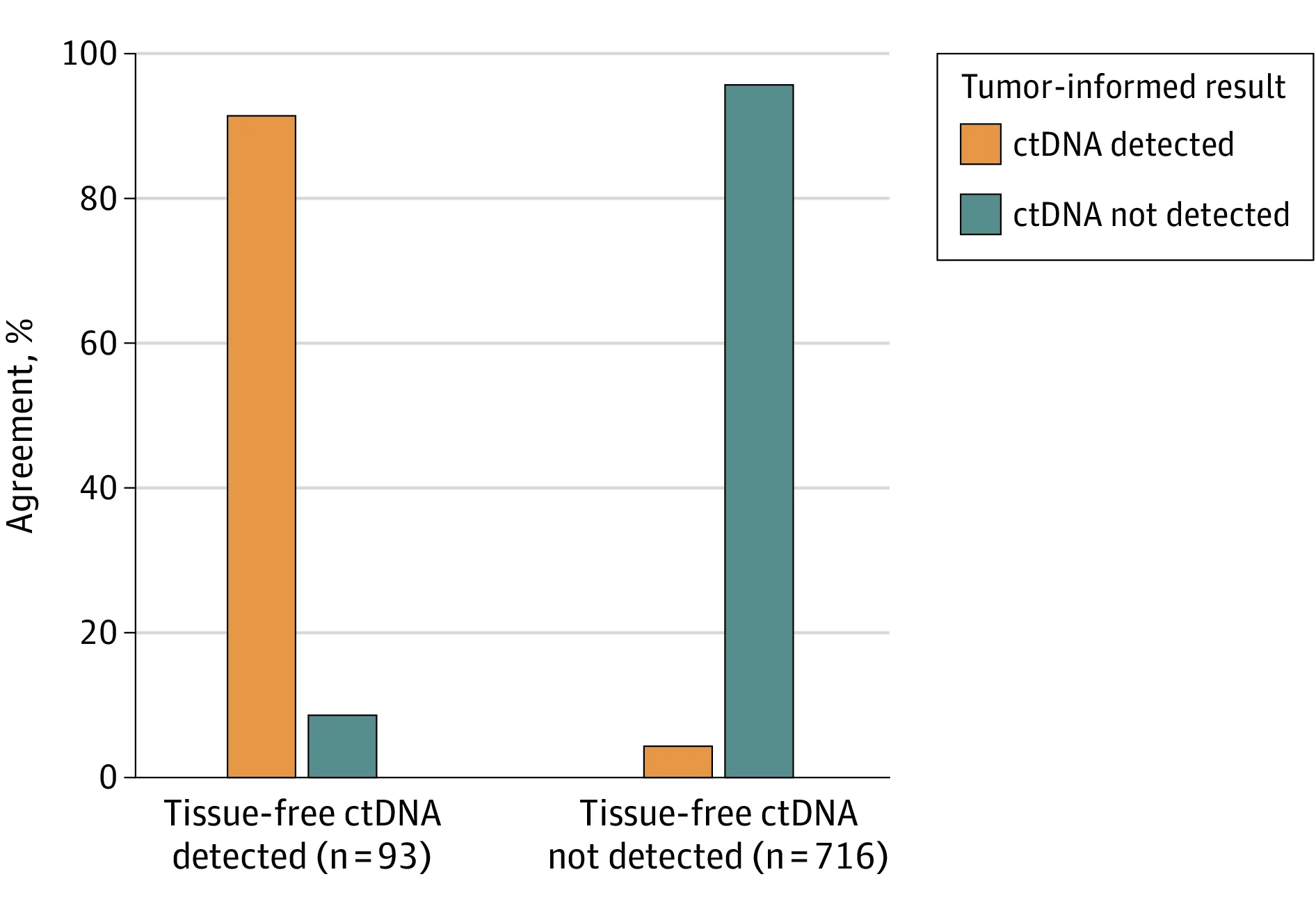
Bar Graph of Comparison Between Tissue-Free and Multivariant Tumor−Informed Detection Results for Circulating Tumor DNA (ctDNA) Of the total samples (n = 809), 85 were concordant positive and 685 were concordant negative, with an overall test agreement of 95.2% (95% CI, 93.5%-96.5%).

Results from both the tissue-free and multivariant tumor-informed assays were available for 133 patients across 809 time points (per patient, median [range], 6 [1-10]). Concordant results were observed in 770 of 809 samples, producing an overall agreement rate of 95.2% (95% CI, 93.5%-96.5%; Cohen κ 0.79 [95% CI, 0.72-0.85]; Figure 4). Of the 93 samples in which ctDNA was detected by the tissue-free assay, 85 (91.4%) were also detected by the multivariant tumor-informed assay. Among the 716 samples classified as ctDNA not detected by the tissue-free assay, 685 (95.7%) were also not detected by the multivariant tumor-informed assay (Figure 4).

**Figure 5.**
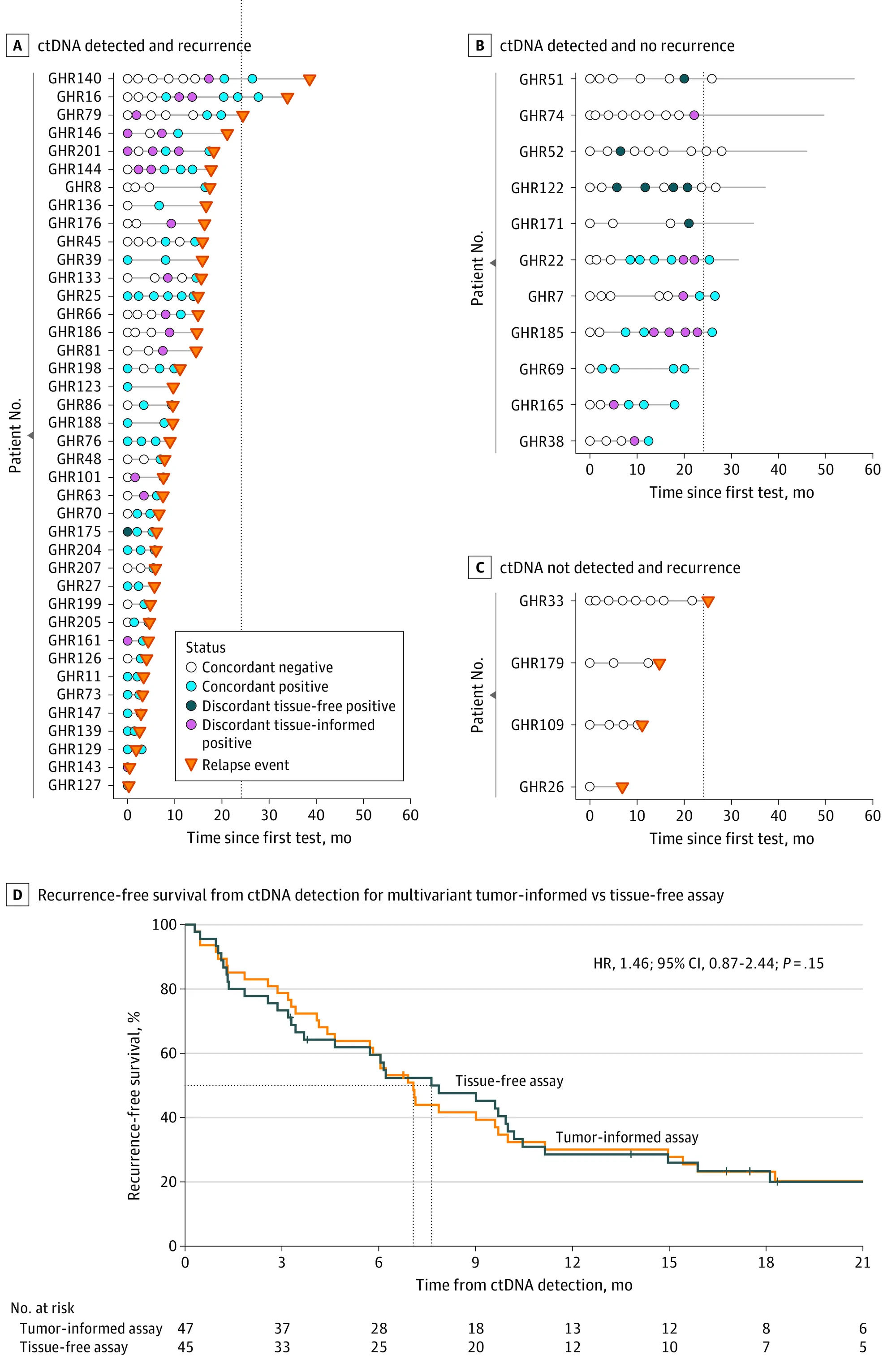
Swimmer Plots and Kaplan-Meier Curve Comparing Tissue-Free to Multivariant Tumor-Informed Detection for Circulating Tumor DNA (ctDNA) A-C, Swimmer plots showing longitudinal data from patients with time points analyzed by both assays. Not shown are 78 patients who did not have ctDNA detected and did not experience recurrence during follow-up. Plots are split by ctDNA detection and recurrence status. D, Kaplan-Meier curve of recurrence-free survival from ctDNA detection for multivariant tumor-informed vs tissue-free assay. The dotted line indicates median recurrence-free survival from ctDNA detection. GHRs are the anonymized patient identifiers. HR indicates hazard ratio.

Overall agreement at a patient level was 123 of 133 (92.5%; 95% CI, 86.6%-96.3%). Among all patients, 41 (30.8%) had ctDNA detected by both assays, 82 (61.7%) did not have ctDNA detected by either assay, while 10 (7.5%) had discordant results. Of 41 patients with ctDNA detected by both assays, 28 (68.3%) were initially detected by both assays in the same time point, 12 (29.3%) were detected earlier by the multivariant tumor-informed assay and 1 (2.4%) was detected earlier by the tissue-free assay (Figures 5A-C). Four patients (9.1% of patients who experienced relapse) experienced clinical relapse without prior detection of ctDNA by either assay. Six patients had ctDNA detected by both tissue-free and multivariant tumor-informed assays in multiple time points but did not experience clinical relapse at data cut-off (Figure 5B). Of the 3 patients with central nervous system only relapse, 2 patients had ctDNA detected prior to relapse which were detected by both assays.

Of the 10 patients with discordant results, 4 had ctDNA detected by only the tissue-free assay and 6 patients, only detected by the multivariant tumor-informed assay (Figures 5A and B). During follow-up, 5 of 6 patients with ctDNA detected by the multivariant tumor-informed assay experienced clinical relapse, whereas none of the patients detected by the tissue-free assay experienced relapse (Figures 5A and B). Characteristics of patients with discordant and concordant results are described in eTable 2 in Supplement 1. In patients with ctDNA detected only by multivariant tumor-informed assay, the median (IQR) estimated allele fraction was 0.002% (0.001%-0.003%) compared to 0.023% (0.005%-0.080%) in patients detected by both assays (*P* = .01, Mann-Whitney U test; eFigure 8 in Supplement 1). In patients with ctDNA detected by only tissue-free assay, the median (IQR) tumor fraction was 0.046% (0.039%-0.051%) compared to 0.113% (0.034%-0.360%) in patients with ctDNA detected by both assays (*P* = .20, Mann-Whitney U test; eFigure 9 in Supplement 1).

The estimated rate of ctDNA detection by 12 months after completion of treatment (date of surgery for neoadjuvant chemotherapy, date of last cycle of chemotherapy for adjuvant chemotherapy) was 33.2% (95% CI, 24.5%-40.8%), with the multivariant tumor-informed assay, and 29% (95% CI, 20.6%-36.5%) with the tissue-free assay (eFigure 10 in Supplement 1). In patients who had ctDNA detected prior to clinical recurrence, the median lead time to relapse was 7.1 (95% CI, 5.7-10.0) months with the multivariant tumor-informed assay and 7.6 (95% CI, 4.6-10.5) months with the tissue-free assay (HR, 1.46; 95% CI, 0.87-2.44; *P* = .15, mixed-effects model; Figure 5D).

## Discussion

A tissue-free approach has the potential to streamline clinical workflows and offer a practical alternative when an archival tumor sample is not available or inadequate to generate tumor-informed assays. However, the potential clinical utility of a tissue-free approach depends on achieving sufficient accuracy and high analytical and clinical sensitivity for ctDNA detection to support clinical decision-making. In this analysis, we leveraged samples from prospective ctDNA surveillance in TNBC and demonstrated that tissue-free ctDNA detection has clinical validity, identifying recurrence with high accuracy. Tissue-free ctDNA detection had a similar lead time to a highly sensitive multivariant tumor-informed approach; however, the multivariant tumor-informed approach detected ctDNA at an earlier time point in a higher proportion of patients.

MRD surveillance with the tissue-free approach was strongly prognostic for disease recurrence with detection anticipating clinical relapse by 7.9 months, comparable to lead times reported in studies using multivariant tumor-informed approaches.^1^ Consistent with previous reports, most ctDNA detections occurred by 12 months of surveillance, reflecting TNBC biologic mechanisms.^18^ All patients in whom ctDNA was detected before adjuvant capecitabine or during standard noncapecitabine chemotherapy, experienced relapse, suggesting that alternative treatments to cytotoxic chemotherapy may be required to improve outcomes for these patients.

In the cTRAK trial,^16^ there was a high incidence of metastatic disease in patients in whom ctDNA was detected by dPCR, emphasizing the need for more sensitive assays than those used prospectively in the study. Tissue-free ctDNA detection occurred at earlier time points compared with dPCR, with a longer lead time to relapse. We hypothesize that the use of this tissue-free assay in cTRAK may have resulted in a greater proportion of patients in whom ctDNA was detected before clinical metastasis development, although additional studies are required to demonstrate this. Furthermore, in 35 of 208 patients (16.8%) cTRAK screening failed due to a lack of archival tissue or dPCR assay design.^16^ These patients would miss clinical testing, and this has both a cost and time implication, highlighting the potential logistical advantages of tissue-free approaches in clinical trials.

To our knowledge, this study is among the first to robustly compare tissue-free ctDNA detection with a multivariant tumor-informed approach. The WES-powered multivariant tumor-informed assay used is a highly sensitive assay, with LOD close to recently developed whole genome sequencing (WGS)−powered assays.^17,20^ There was good concordance between tissue-free and multivariant tumor-informed assays at a sample and patient level. Nevertheless, more patients had ctDNA initially detected at an earlier time point by the multivariant tumor-informed assay than by the tissue-free assay. Moreover, a small number of patients who experienced relapse only had ctDNA detected by the tumor-informed assay, suggesting its superior analytical sensitivity. However, the increased analytical sensitivity of the multivariant tumor-informed assay did not translate into a longer median lead time between detection and clinical recurrence, suggesting that the increased analytical sensitivity of the multivariant tumor-informed assay may not make a substantial difference in the lead time of MRD monitoring studies.

Studies in which ctDNA analysis is used to select patients for treatment deescalation require highly sensitive assays to avoid withholding treatment from patients without ctDNA detection who subsequently experience recurrence. Recent studies in colorectal cancer, such as the DYNAMIC-III trial^21^ were unable to demonstrate noninferiority of ctDNA-guided treatment deescalation in stage III disease, partly because the assay used may have had inadequate sensitivity to detect micrometastatic disease with low shedding of ctDNA. Our study utilized samples from a treatment escalation study to demonstrate that the lead times from detection to recurrence were comparable between the assays; however, we would suggest caution implementing the tissue-free approach in de-escalation studies in early breast cancer, unless it is not possible to generate a tumor-informed assay. More broadly, the clinical utility of MRD detection and escalating intervention remains an important unanswered question to be addressed in ongoing studies such as TRAK-ER (NCT04985266) and DARE (NCT04567420).

Detection of ctDNA in patients who do not subsequently experience relapse has been reported in MRD studies across tumor types.^4,22,23^ Distinguishing false positives from ctDNA detection at low levels representative of immune surveillance of micrometastatic disease, or long lead times in patients with insufficient clinical follow-up is challenging. In our study, 6 patients had ctDNA detected by both tissue-free and multivariant tumor-informed assays at multiple time points. The concordant results support the hypothesis that in a proportion of patients, ctDNA detection may represent a degree of immune surveillance or more indolent TNBC biologic mechanisms, with insufficient follow-up to capture clinical recurrence. Studies are required to characterize these patients and improve our understanding of the biologic mechanisms of cancer in these patients. Furthermore, patients did not have routine surveillance imaging to assess for metastatic disease in the absence of symptoms aligned with standard guidelines^24^; therefore, it is not known whether these patients had occult metastatic disease at the time of detection, potentially introducing bias in the assessment of lead time. In the comparison between tissue-free and multivariant tumor informed approaches, none of the 4 patients with ctDNA detected only by the tissue-free assay experienced relapse during follow up. We are unable to address whether these patients would experience relapse during a longer follow-up period; represent true ctDNA detection that cleared, perhaps due to immune surveillance; represent detection of a different cancer; or had false-positive results.

### Limitations

A potential limitation of this study is that we did not compare the tissue-free to WGS-powered tumor-informed assays. However, to our knowledge, the multivariant tumor-informed assay used in our study is among the most sensitive WES-based tumor-informed approaches (LOD_95_ of 0.0011% allele fraction equivalent to approximately 11 parts per million),^17^ with analytical sensitivity approaching that of WGS-powered assays (LOD_95_ of 3.45 parts per million),^20^ which reflects optimization of the assay workflow, including tracking up to 48 variants per assay.^17^ It is possible that the median lead time of a WGS-powered ctDNA assay may be longer than we observed in this study. Efforts to further improve the analytical sensitivity of tissue-free assays may optimize ctDNA detection to guide treatment decisions.

## Conclusions

In this prognostic study, tissue-free ctDNA detection during surveillance was strongly prognostic for recurrence of TNBC, demonstrating clinical validity in this setting. The median lead time of the tissue-free ctDNA assay was similar to a multivariant tumor-informed assay, although the tumor-informed approach detected ctDNA at earlier time points in a greater proportion of patients. These findings support the use of tissue-free assays as a practical alternative in patients for which a tumor-informed assay is not possible. However, the clinical benefit of early MRD detection and intervention remains uncertain and prospective ctDNA-guided intervention studies are warranted to assess clinical utility of tissue-free assays.

## Notes

Supplement 1. eMethods eFigure 1. CONSORT Diagram eFigure 2. Tumor methylation fraction at first timepoint detection by clinical risk group in patients with ctDNA detected by the tissue-free assay eFigure 3. Tumor methylation fraction at first timepoint detection by recurrence status in patients with ctDNA detected by the tissue-free assay eFigure 4. Tumor methylation fraction at first ctDNA detection vs lead time to recurrence in patients with ctDNA detected by the tissue-free assay eFigure 5. Tissue-free ctDNA detection in Triple Negative Breast cancer (TNBC) eFigure 6. Diagnostic performance of the tissue-free assay for recurrence at any time during follow-up eFigure 7. Comparison of Volumes Sent for Analysis by Tissue-free and Tumor-informed Assays eFigure 8. Estimated allele fraction (eVAF) at first ctDNA detection by detection status in patients with paired tissue-free and multivariant tumor-informed analysis eFigure 9. Tumor methylation fraction at first ctDNA detection by detection status in patients with paired tissue-free and multivariant tumor-informed analysis eFigure 10. Time from start of ctDNA surveillance to ctDNA detection. eTable 1. Table demonstrating the clinical characteristics of patients on surveillance in c-TRAK TN eTable 2. Table of characteristics of concordant and discordant detection groups in the comparison between tissue-free and tumor-informed assays

Supplement 2. Data Sharing Statement
