# Supplementary material for: Tissue-Free vs Tumor-Informed ctDNA Assays for Molecular Residual Disease Detection in Early Triple Negative Breast Cancer
Source: JAMA Oncol. 2026 Aug 13;12(10):1098–106. doi: 10.1001/jamaoncol.2026.2833 (PMC13474100; doi:10.1001/jamaoncol.2026.2833)
Supplement: Supplementary file 1 [file jamaoncol-e262833-s001.pdf]

# Supplemental Online Content

Cunningham N, Cutts RJ, Swift C, et al. Tissue-free vs tumor-informed ctDNA assays for molecular residual disease detection in early triple negative breast cancer. *JAMA Oncol*. Published online August 13, 2026. doi:10.1001/jamaoncol.2026.2833

**eMethods**

**eFigure 1.** CONSORT Diagram

**eFigure 2.** Tumor methylation fraction at first timepoint detection by clinical risk group in patients with ctDNA detected by the tissue-free assay

**eFigure 3.** Tumor methylation fraction at first timepoint detection by recurrence status in patients with ctDNA detected by the tissue-free assay

**eFigure 4.** Tumor methylation fraction at first ctDNA detection vs lead time to recurrence in patients with ctDNA detected by the tissue-free assay

**eFigure 5.** Tissue-free ctDNA detection in Triple Negative Breast cancer (TNBC)

**eFigure 6.** Diagnostic performance of the tissue-free assay for recurrence at any time during follow-up

**eFigure 7.** Comparison of Volumes Sent for Analysis by Tissue-free and Tumor-informed Assays

**eFigure 8.** Estimated allele fraction (eVAF) at first ctDNA detection by detection status in patients with paired tissue-free and multivariant tumor-informed analysis

**eFigure 9.** Tumor methylation fraction at first ctDNA detection by detection status in patients with paired tissue-free and multivariant tumor-informed analysis

**eFigure 10.** Time from start of ctDNA surveillance to ctDNA detection.

**eTable 1.** Table demonstrating the clinical characteristics of patients on surveillance in c-TRAK TN

**eTable 2.** Table of characteristics of concordant and discordant detection groups in the comparison between tissue-free and tumor-informed assays

This supplemental material has been provided by the authors to give readers additional information about their work.

© 2026 Cunningham N, et al. JAMA Oncol.

**eMethods**

**Clinical Risk Stratification**

Patients were stratified into high and moderate risk of recurrence group defined in the c-TRAK TN protocol.

For patients who had received neoadjuvant chemotherapy with no adjuvant chemotherapy planned:

- High risk: residual microscopic or macroscopic invasive cancer in the axillary nodes after chemotherapy
- Moderate risk: residual invasive cancer in the breast and axillary lymph node negative after chemotherapy

For patients who had received adjuvant chemotherapy

- High risk: tumor size >50mm and node positive OR ≥4 nodes positive regardless of primary tumor size.
- Moderate risk: tumor size ≥20mm AND/OR involved axillary macroscopic lymph node

Patients who received both neoadjuvant and adjuvant chemotherapy were stratified as per the adjuvant chemotherapy alone cohort on either clinical or pathological staging.

**Imaging and Follow-Up**

Imaging was performed as per the c-TRAK TN protocol. Clinical recurrence date was defined as the date of radiological evidence of recurrence.

During ctDNA surveillance, imaging was not mandated within the trial protocol and was guided by clinical assessment. Patients were followed up for recurrence in line with standard practice every 6 months (+/- 1 month) from start of ctDNA surveillance. Post ctDNA surveillance this could be conducted by telephone call to the patients.

© 2026 Cunningham N, et al. JAMA Oncol.

Patients who had ctDNA detected and were randomized to receive pembrolizumab had imaging prior to starting pembrolizumab using the same imaging used for staging at diagnosis or in line with local guidelines. Additional imaging was not mandated within the trial protocol and was guided by clinical assessment. Patients were followed up for recurrence in line with standard practice every 6 months (+/-1 month) from start of ctDNA surveillance. After 16 June 2020 (Protocol V6) all patients with ctDNA detected were allocated to pembrolizumab, and those that were still under ctDNA surveillance were allocated to receive pembrolizumab at the next ctDNA detection.

Patients who had ctDNA detected and were randomized to observation, imaging was guided by clinical assessment. Patients were followed up for recurrence in line with standard practice every 6 months (+/-1 month) from start of ctDNA surveillance. Post ctDNA surveillance this was permissible by telephone call to the patients.

**Potential Impact of Scanning on Lead Time Estimates**

The protocol did not mandate scanning during surveillance or follow-up consistent with international guidelines and contemporaneous clinical practice. Given the translational nature of this analysis, there is a potential risk of bias associated with the calculation of lead time. In most cases, decision to scan patients was triggered by the reporting of symptoms, therefore biological recurrence may predate the recorded time of recurrence. The site of metastatic disease may also influence pattern of symptoms reported, and thus timing of recurrence detection. This limitation is the same across all assays thus the comparisons across these assays are valid, however the true lead time may differ from that reported here and should be interpreted with caution. Detection of ctDNA by dPCR was actionable within the study and may introduce bias, particularly in the comparison between dPCR and tissue-free assay.

**Tissue-Free Methylation Based Assay**

The tissue-free assay, Reveal (Guardant Health, Palo Alto, CA) utilizes a custom hybrid-capture panel (Twist Biosciences, South San Francisco, CA) targeting >20,000 differentially methylated regions (DMRs) associated with ctDNA, including 3000 breast specific regions[1]. Target regions were selected using public methylation

© 2026 Cunningham N, et al. JAMA Oncol.

datasets, including TCGA and the Blueprint Project, and internally generated cancer and non-cancer reference datasets[2]. The assay was designed to provide broad genomic coverage of regulatory regions while excluding loci with consistently methylated or unmethylated patterns in normal tissues to improve tumor specificity. cfDNA was extracted from 1.5 to 4 mL of plasma and partitioned by methylation state. Methylated cfDNA was ligated with unique molecular barcodes and amplified by polymerase chain reaction (PCR). Libraries were enriched and sequenced on an Illumina NovaSeq 6000. Methylation signal was quantified by normalizing molecular coverage across targeted regions relative to matched controls. ctDNA detection and tumor methylation fraction were derived from region-level methylation patterns using regression-based models trained on cancer-specific and pan-cancer cohorts comprising approximately 5,000 clinical samples[3]. Thresholds for MRD detection were established using an independent validation cohort of >5,000 negative control samples.

**Whole exome based Multivariant Tumor-Informed Assay**

Patients required primary tumor tissue, buffy coat and plasma from at least one timepoint for analysis with the multivariant tumor-informed assay. Briefly, RaDaR (NeoGenomics Laboratories, Inc., Fort Myers, FL) is a patient-personalized assay that utilizes multiplex PCR and targeted next generation sequencing (NGS) to detect ctDNA in plasma and achieve a limit of detection of 0.0011%[4]. Whole exome sequencing was performed on tumor and matched buffy coat samples. Custom primer panels were designed using proprietary algorithms and included a median of 43 variants (range 9-61). cfDNA was extracted from at least 2 mL of plasma and analyzed using the RaDaR assay. Presence of ctDNA was determined using proprietary bioinformatic methods with a tumor fraction generated for each sample.

**dPCR Assay**

Patient personalized dPCR assays were utilized for prospective ctDNA analysis in c-TRAK TN as previously published[5,6]. DNA was extracted from archival FFPE tumor samples and subjected to targeted panel sequencing with fixed gene panels. One or two variants were selected per patient for dPCR assay design. Successful validation required the assay to be positive in two tissue samples and negative in DNA extracted from matched buffy coat. cfDNA was extracted from 4 mL of plasma and

© 2026 Cunningham N, et al. JAMA Oncol.

analyzed by dPCR on a Bio-Rad QX200 instrument (BioRad, Watford Hertfordshire). ctDNA was detected if at least two mutant (FAM) droplets were identified using previously established criteria[7]. Copies per mL and allele fraction were calculated as previously described[7].

© 2026 Cunningham N, et al. JAMA Oncol.

## Summary of Assays Utilized in the Comparison

| Assay | Type | Platform | Variants / Signals Tracked | Limit of Detection (LOD) | Tumor Tissue Requirement |
|---|---|---|---|---|---|
| **dPCR** | Tumor-informed | Droplet digital PCR (dPCR) | 1-2 patient-specific variants | 1-3 mutant molecules per 10000 (~0.01%) [7] | Yes |
| **RaDaR** | Multivariant tumor-informed | Personalized multiplex PCR + targeted deep NGS + error suppression (NeoGenomics/Inivata RaDaR) | Up to ~48 patient-specific variants | $LOD_{95}$ of 0.0011% allele fraction equivalent to ~11 PPM[4] | Yes |
| **Guardant Reveal** | Tissue-free | Hybrid capture NGS with methylation analysis | >20,000 differentially methylated regions (3000 breast specific) | $LOD_{95}$ of 0.02% with 3-4 ng cfDNA input and 0.005% with 15 ng input equivalent to ~50 PPM[3] | No |

## Statistical Analysis

Survival endpoints were analyzed using Kaplan-Meier method. Recurrence free survival (RFS) was taken from the start of surveillance to recurrence of breast cancer or death. Patients who did not have recurrence or death were censored at last follow-up. RFS by detection of ctDNA at any timepoint using the tissue-free assay was assessed using a time-dependent Cox regression model. Time to ctDNA detection was defined from the start of ctDNA surveillance to first ctDNA detected result, with patients censored at discontinuation of ctDNA surveillance. Hazard ratios (HRs) and 95% confidence intervals (95% CI) were calculated within Cox proportional hazards regression models. The proportion of patients who had ctDNA detected by 12 and 24 months from the start of ctDNA surveillance was estimated from the Kaplan-Meier curve. The median lead time was calculated from the date

© 2026 Cunningham N, et al. JAMA Oncol.

ctDNA was first detected to the date of clinical recurrence, censoring at date of withdrawal or date of last follow-up and reported for each assay. A mixed effects Cox regression with patient ID as a random effect was used to compare lead time between assays. To assess diagnostic accuracy of the tissue-free assay, we calculated sensitivity for recurrence by 24 months. This analysis included patients who had clinical recurrence by 24 calendar months after the start of surveillance and those who were recurrence-free at 24 months. Median follow-up was calculated using reverse Kaplan-Meier method. The threshold for statistical significance was defined as 0.05 with two-sided testing. Statistical analysis was performed with R statistical software version 4.2.2.

## References


1. Elliott MJ, Kim J, Dou A, et al. Comprehensive tumor-agnostic evaluation of genomic and epigenomic-based approaches for the identification of circulating tumor DNA in early-stage breast cancer. *ESMO Open*. 2025;10(6)doi:10.1016/j.esmoop.2025.105286
2. Gross A, Zhao J, Quinn K, et al. Abstract 5936: Landscape of epigenomic tumor fraction in large pan-cancer cfDNA cohort. *Cancer Research*. 2025;85(8_Supplement_1):5936-5936. doi:10.1158/1538-7445.Am2025-5936
3. Quinn K, Wilfert A, Lakshmin R, et al. Abstract 692: Analytical validation of a tissue-free epigenomic assay for circulating tumor DNA (ctDNA)-based molecular residual disease (MRD) detection in early-stage cancer. *Cancer Research*. 2025;85(8_Supplement_1):692-692. doi:10.1158/1538-7445.AM2025-692
4. Flach S, Howarth K, Hackinger S, et al. Liquid BIOpsy for MiNimal RESidual DiSease Detection in Head and Neck Squamous Cell Carcinoma (LIONESS)—a personalised circulating tumour DNA analysis in head and neck squamous cell carcinoma. *British Journal of Cancer*. 2022/05/01 2022;126(8):1186-1195. doi:10.1038/s41416-022-01716-7
5. Garcia-Murillas I, Schiavon G, Weigelt B, et al. Mutation tracking in circulating tumor DNA predicts relapse in early breast cancer. *Sci Transl Med*. Aug 26 2015;7(302):302ra133. doi:10.1126/scitranslmed.aab0021
6. Turner NC, Swift C, Jenkins B, et al. Results of the c-TRAK TN trial: a clinical trial utilising ctDNA mutation tracking to detect molecular residual disease and trigger intervention in patients with moderate- and high-risk early-stage triple-negative breast cancer. *Ann Oncol*. Feb 2023;34(2):200-211. doi:10.1016/j.annonc.2022.11.005
7. Garcia-Murillas I, Chopra N, Comino-Méndez I, et al. Assessment of Molecular Relapse Detection in Early-Stage Breast Cancer. *JAMA Oncol*. Oct 1 2019;5(10):1473-1478. doi:10.1001/jamaoncol.2019.1838


© 2026 Cunningham N, et al. JAMA Oncol.

## eFigures and Tables (Supplementary)

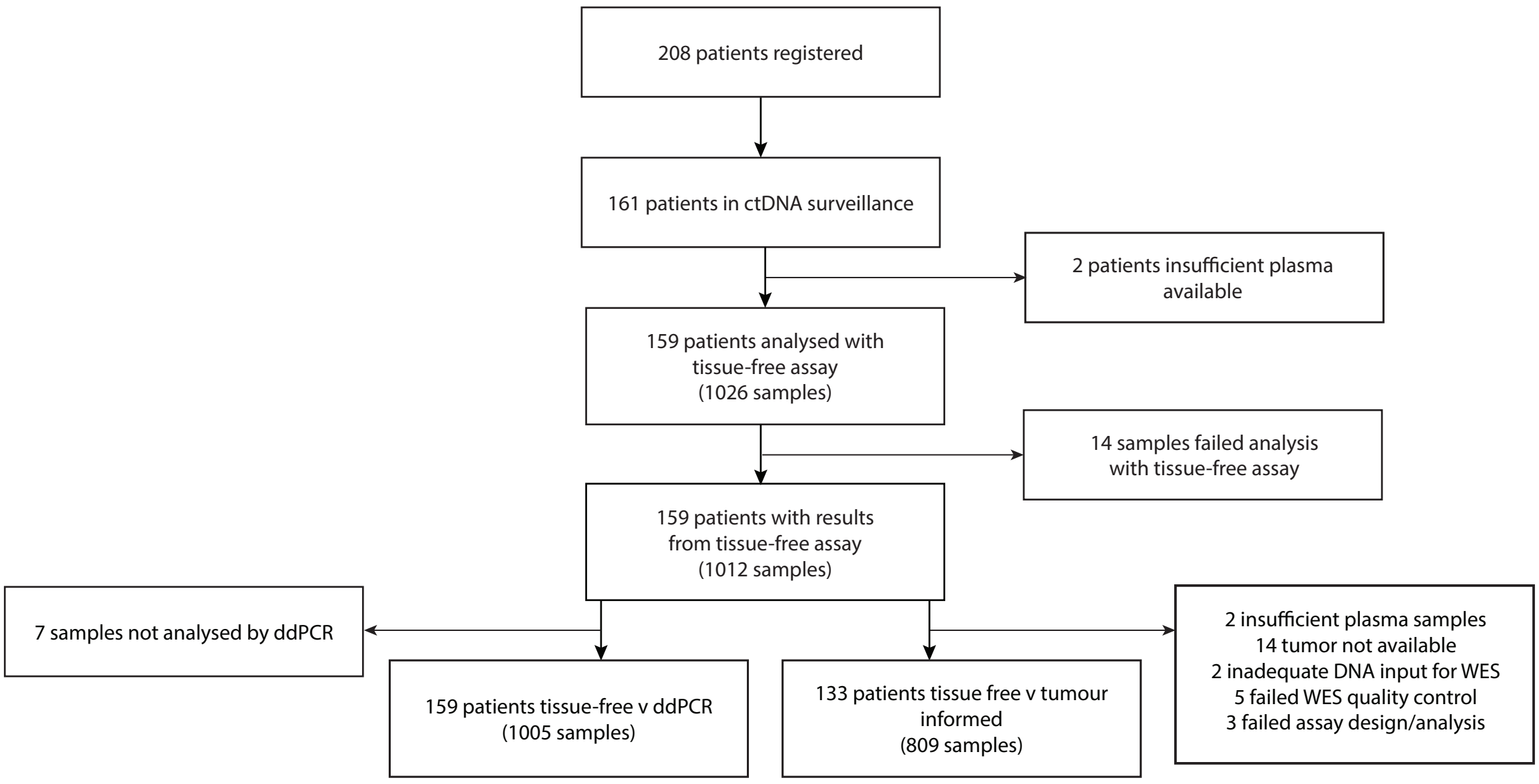


eFigure 1: CONSORT Diagram.

Patients from c-TRAK surveillance were included in this study if they had plasma available from at least 1 timepoint for analysis with the tissue-free assay. Samples failed analysis with tissue-free assay due to low cfDNA yield (n=13) or suspected contamination (n=1). WES = whole exome sequencing.

© 2026 Cunningham N, et al. JAMA Oncol.

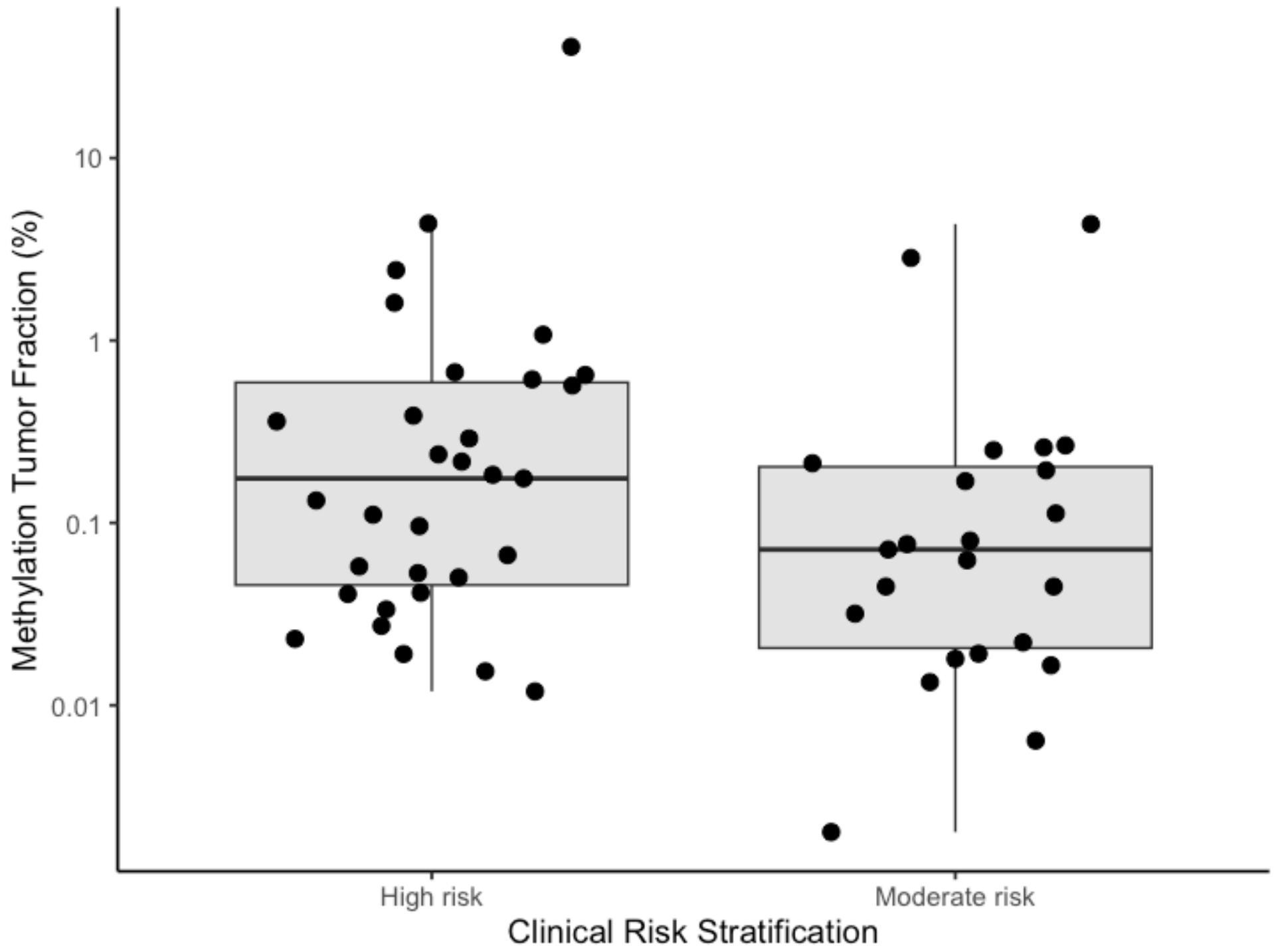


eFigure 2: Tumor methylation fraction at first timepoint detection by clinical risk group in patients with ctDNA detected by the tissue-free assay.

The median tumor methylation fraction was 0.175% (IQR 0.046-0.589%) in patients at high risk of recurrence and 0.072% (IQR 0.021-0.203%) in moderate risk patients (p = 0.08, Mann-Whitney U)

© 2026 Cunningham N, et al. JAMA Oncol.

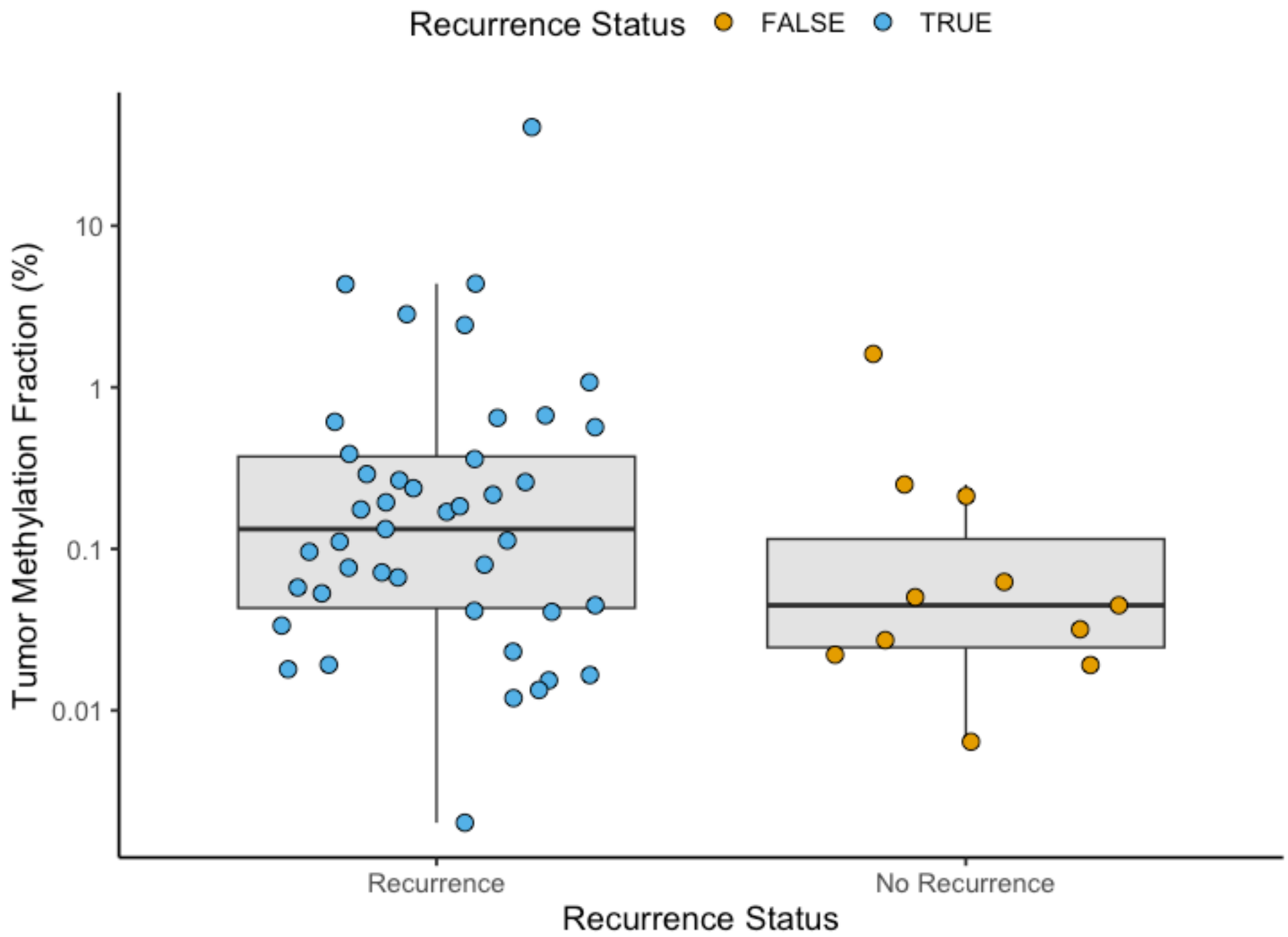


eFigure 3: Tumor methylation fraction at first timepoint detection by recurrence status in patients with ctDNA detected by the tissue-free assay.

The median tumor methylation fraction was 0.133% (IQR 0.043-0.374%) in patients who had a recurrence, compared with 0.045% (IQR 0.024-0.137%) in those who did not (p=0.098, Mann-Whitney U).

© 2026 Cunningham N, et al. JAMA Oncol.

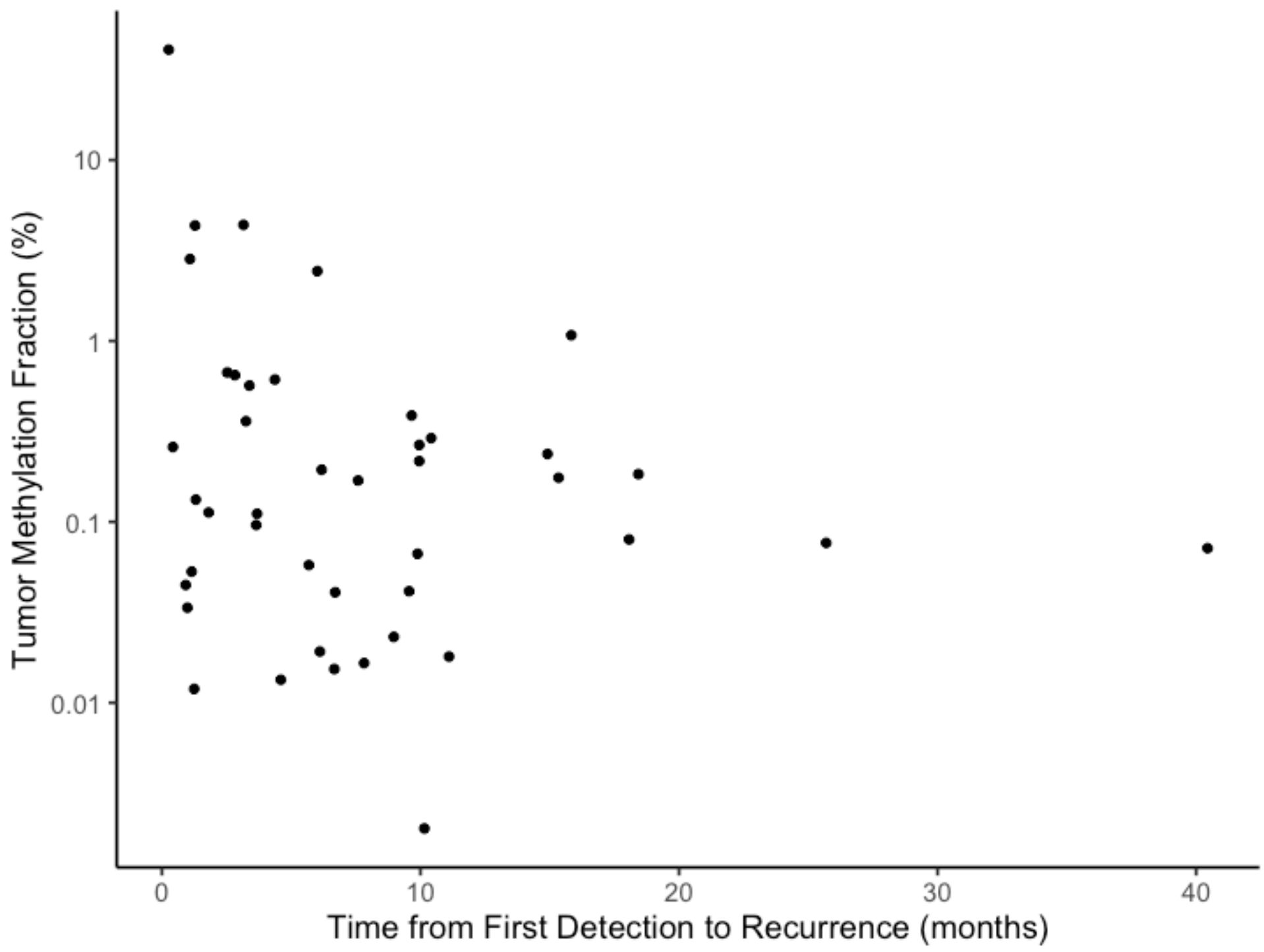


eFigure 4: Tumor methylation fraction at first ctDNA detection vs lead time to recurrence in patients with ctDNA detected by the tissue-free assay.

© 2026 Cunningham N, et al. JAMA Oncol.

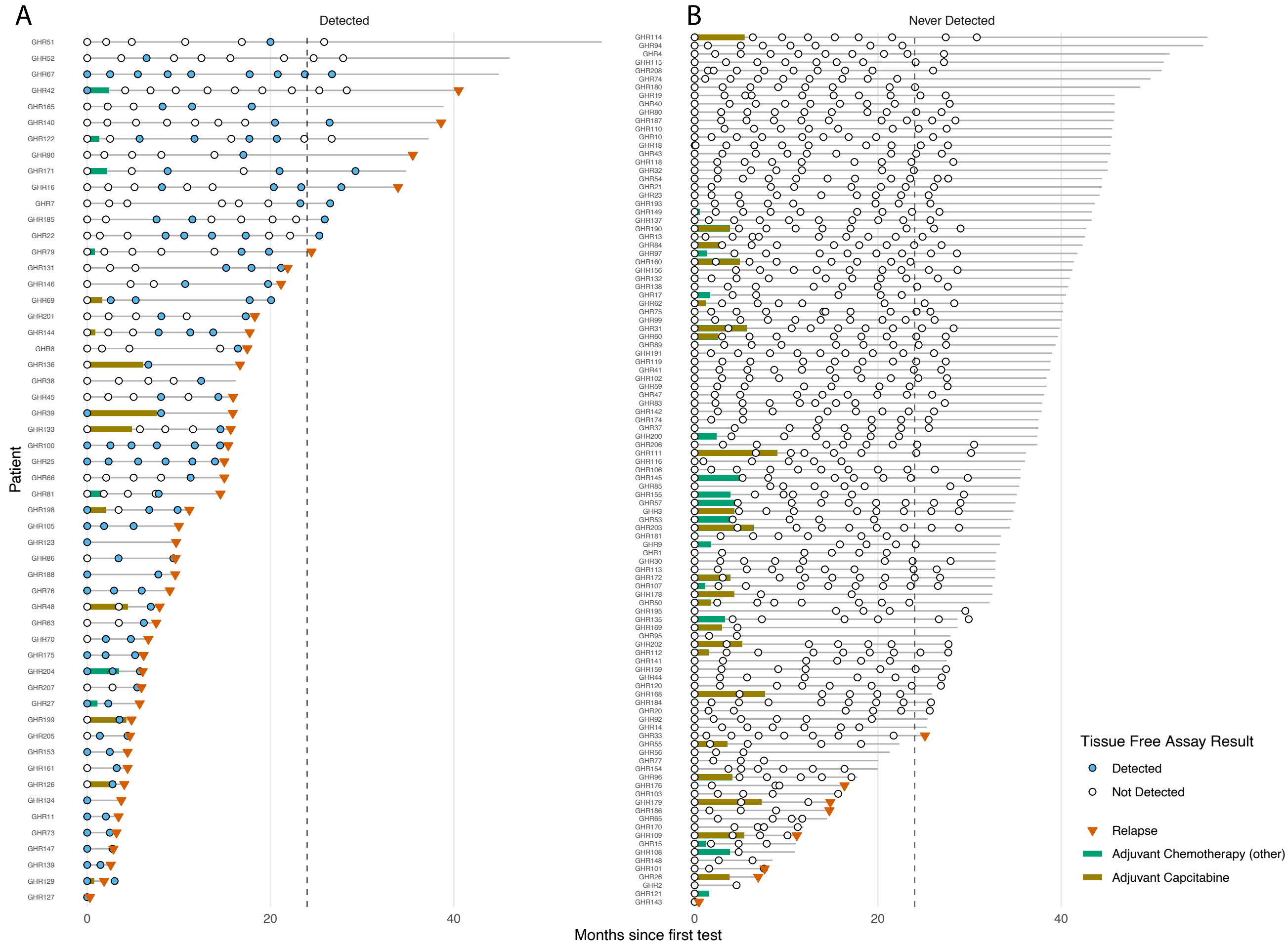


eFigure 5: Tissue-free ctDNA detection in Triple Negative Breast cancer (TNBC).

Longitudinal data from patients with TNBC in the cTRAK study during ctDNA surveillance. Analysis for ctDNA is represented by a circle, blue for ctDNA detected and clear for ctDNA not detected. Inverted orange triangle represents recurrence. Grey line indicates follow-up. Adjuvant chemotherapy received is represented by thick colored line, green for adjuvant chemotherapy other and yellow for adjuvant capecitabine. Patients are divided by detected in at least 1 timepoint (A) and never detected (B).

© 2026 Cunningham N, et al. JAMA Oncol.

| | Recurrence | No Recurrence |
|---|---|---|
| Tissue-free ctDNA detected | 43 | 11 |
| Tissue-free ctDNA not detected | 8 | 97 |

Sensitivity = 43/51=84.3%
Specificity = 97/108 = 89.8%
Positive predictive value = 43/54 =79.6%
Negative predictive value = 97/105=92.4%

eFigure 6: Diagnostic performance of the tissue-free assay for recurrence at any time during follow-up.

© 2026 Cunningham N, et al. JAMA Oncol.

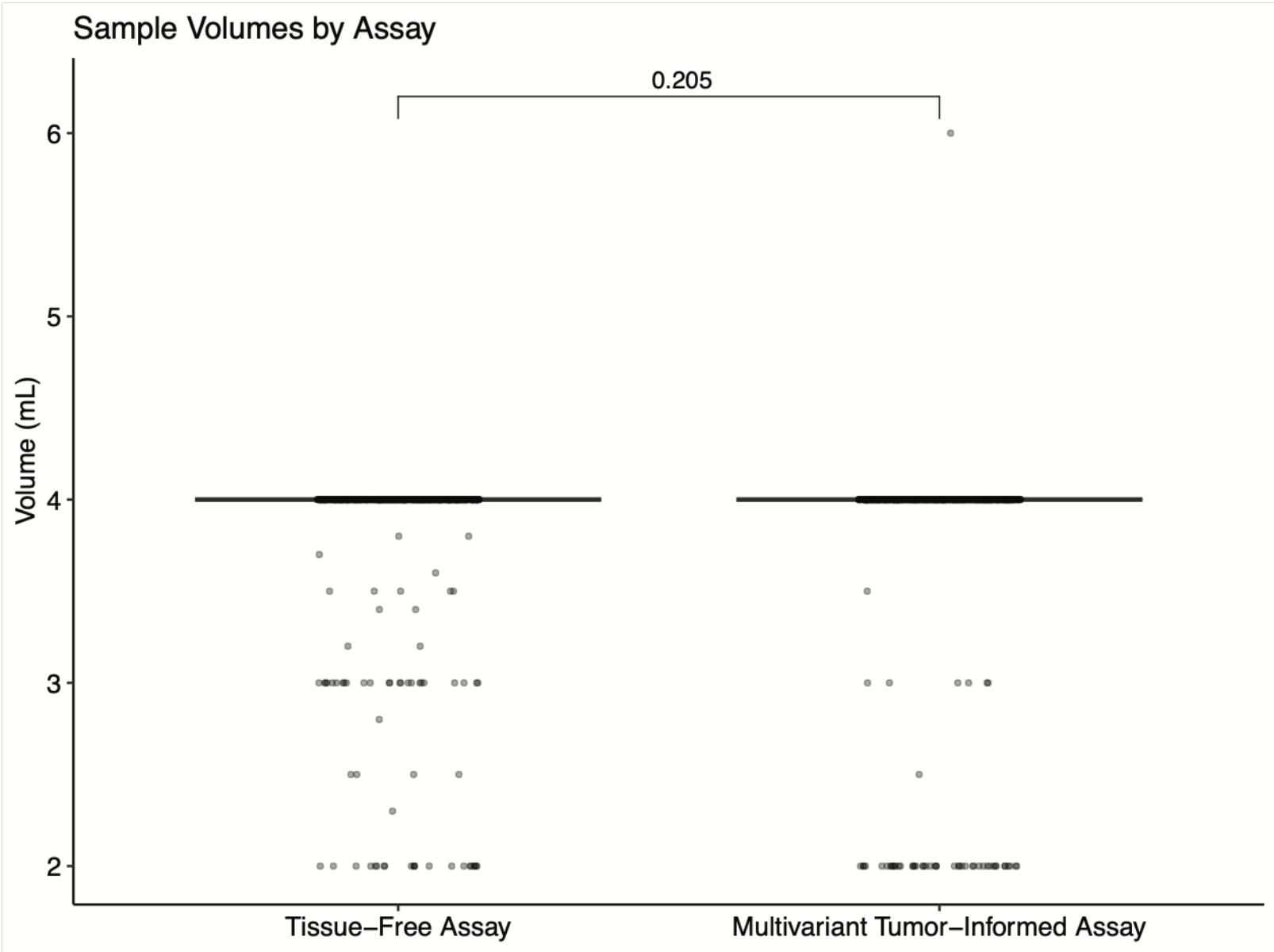


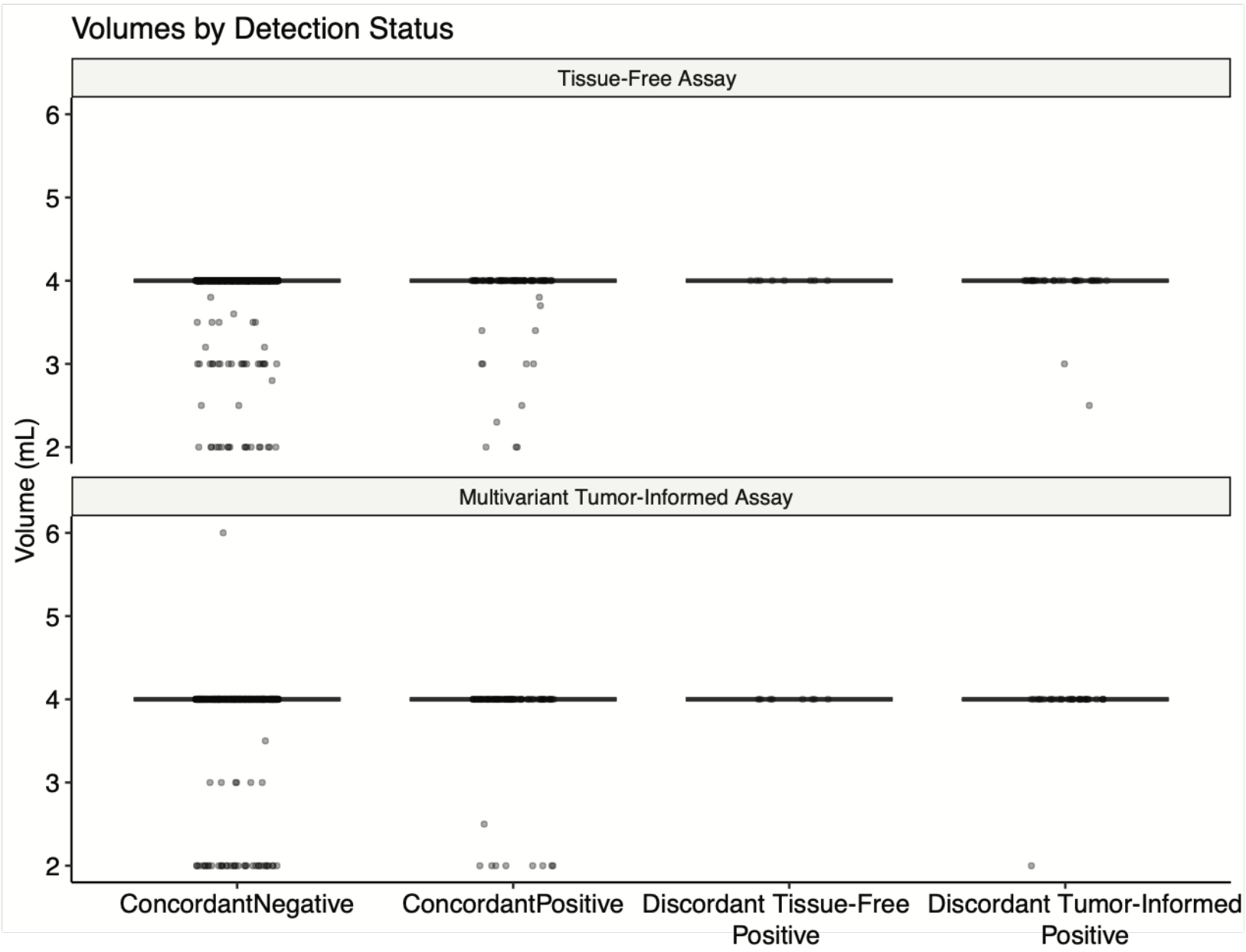


eFigure 7: Comparison of Volumes Sent for Analysis by Tissue-free and Tumor-informed Assays.

The mean volume for the tissue-free assay was 3.89 mL compared with 3.88 mL for the multivariant tumor-informed assay (Wilcoxon rank signed test, p=0.21) (top). There was no statistically significant difference between the volumes analyzed by the tissue-free approach between samples that were detected by both assays and just detected by the tumor-informed approach (bottom).

© 2026 Cunningham N, et al. JAMA Oncol.

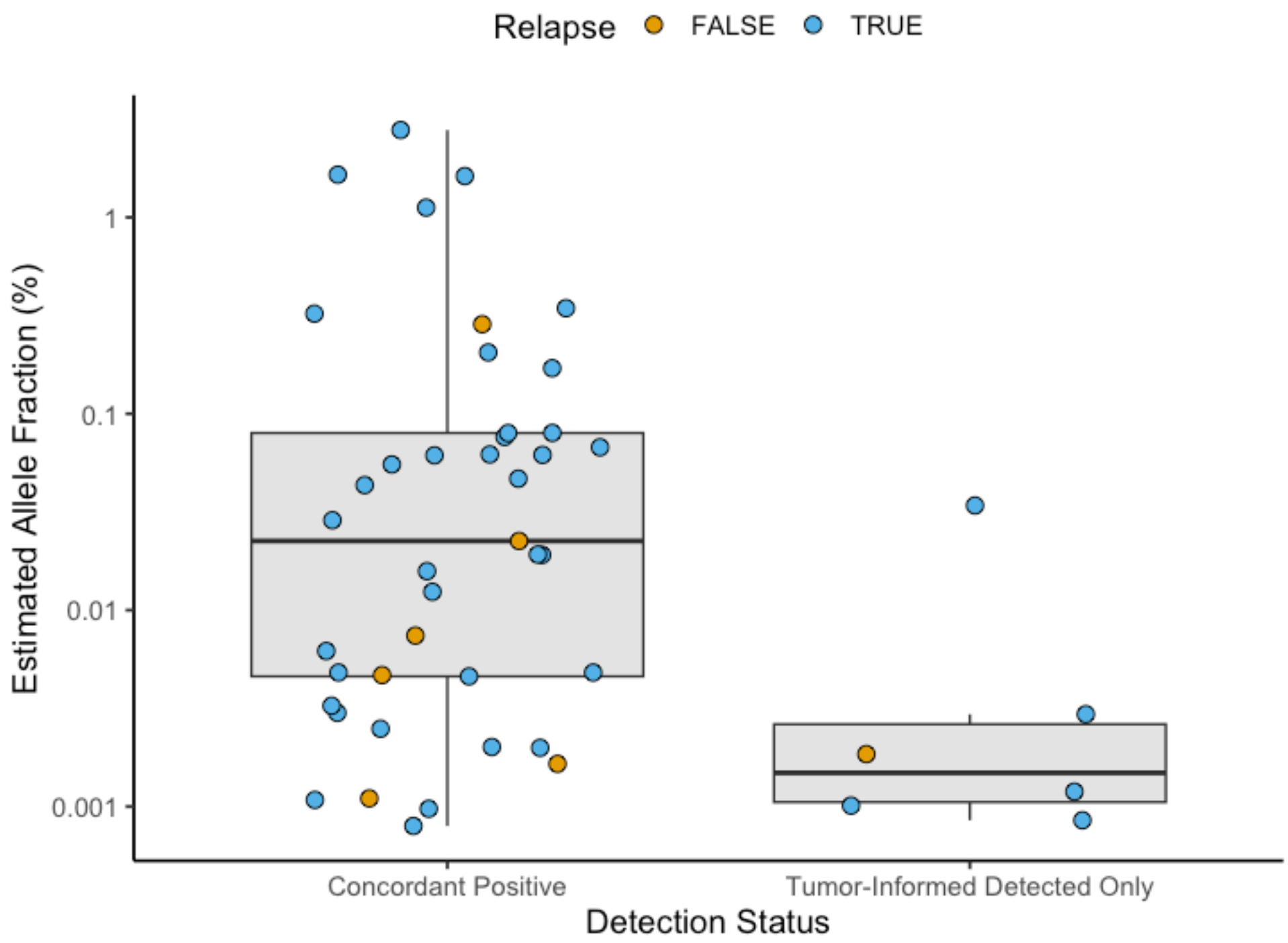


eFigure 8: Estimated allele fraction (eVAF) at first ctDNA detection by detection status in patients with paired tissue-free and multivariant tumor-informed analysis.

The median eVAF was 0.023% (IQR 0.005-0.080%) in patients detected by both assays and 0.002% (IQR 0.001-0.003%) in patients detected by the tumor-informed assay only (p=0.010, Mann-Whitney U).

© 2026 Cunningham N, et al. JAMA Oncol.

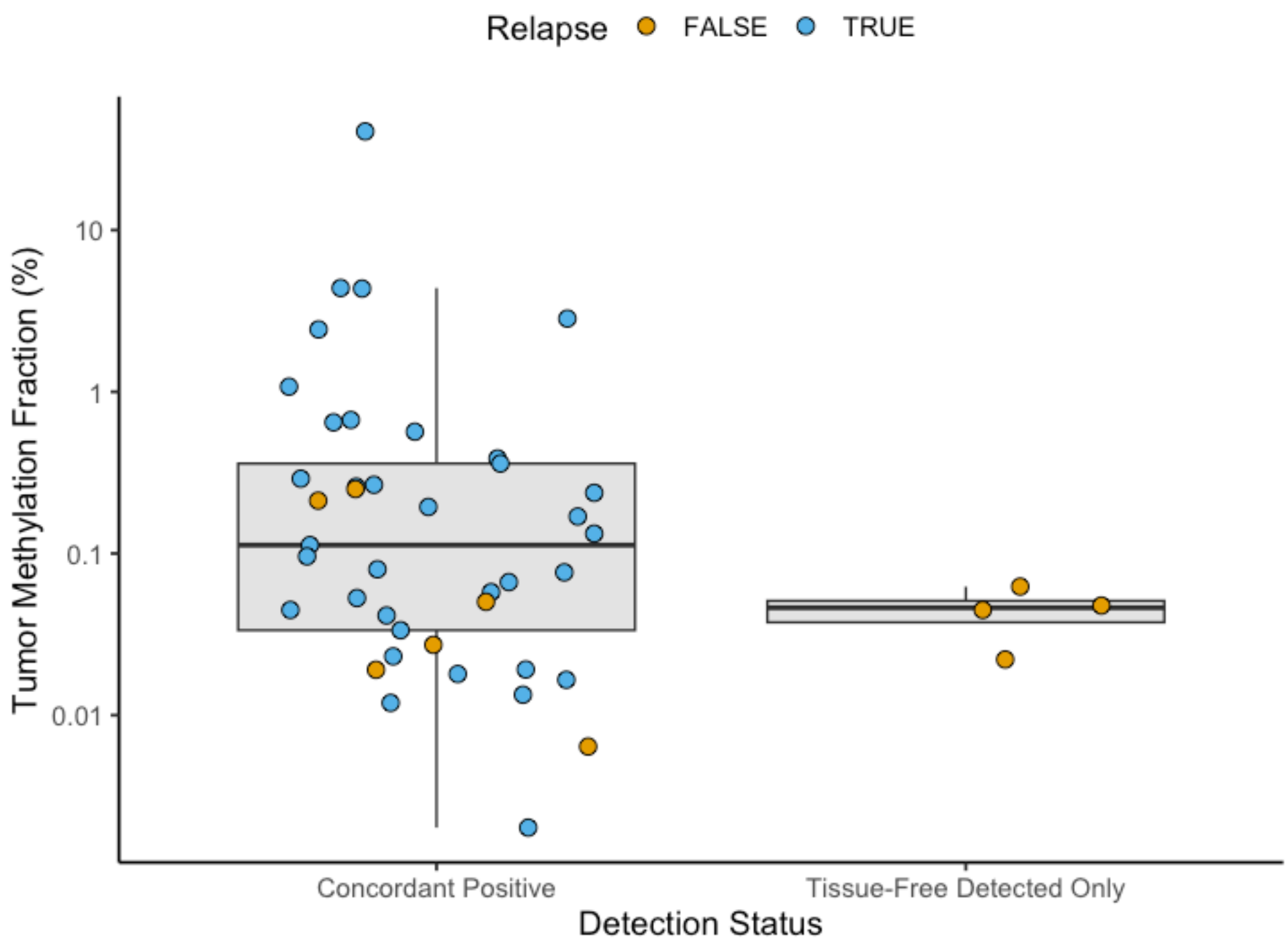


eFigure 9: Tumor methylation fraction at first ctDNA detection by detection status in patients with paired tissue-free and multivariant tumor-informed analysis.

In patients with ctDNA detected only by tissue-free assay the median TF in the first timepoint detected was 0.046% (IQR 0.039-0.051%) compared to 0.113% (IQR 0.034-0.360%) in patients detected by both assays (p=0.203, Mann-Whitney U)

© 2026 Cunningham N, et al. JAMA Oncol.

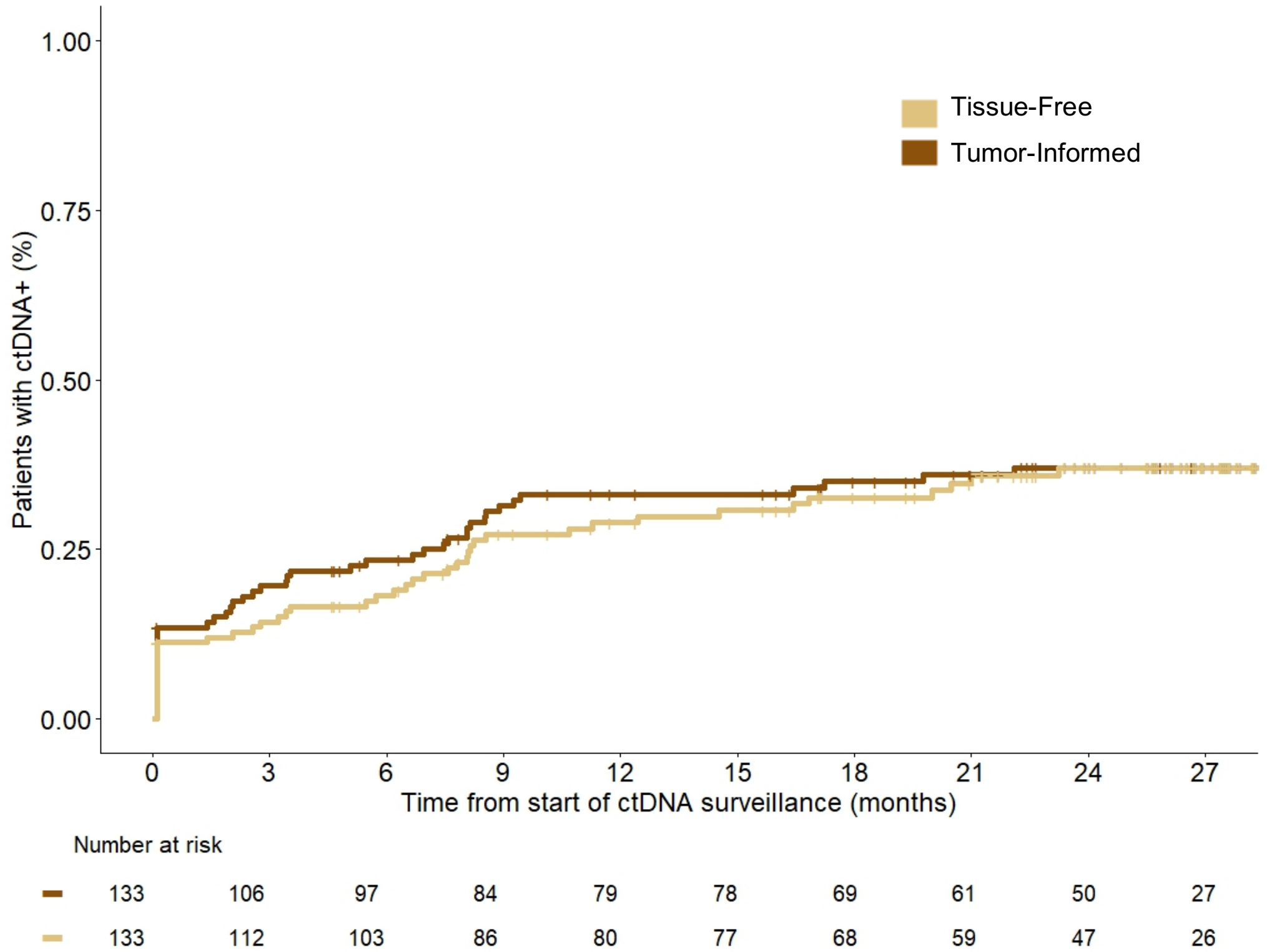


eFigure 10: Time from start of ctDNA surveillance to ctDNA detection.

A comparison between patients with ctDNA detected by the tissue-free assay and multivariant tumor-informed assay (HR 0.94 (95%CI: 0.62 - 1.41) P=.753.

© 2026 Cunningham N, et al. JAMA Oncol.

| | | All c-TRAK TN Surveillance population | | Patients analysed with Tissue-Free Assay | | Patients analysed with Tissue-Free and Multivariant Tumor Informed Assays | |
|---|---|---|---|---|---|---|---|
| | | N=161 | | N=159 | | N=133 | |
| | | n | % | n | % | n | % |
| **Type of definitive surgery** | Mastectomy | 55 | 34.2 | 54 | 34.0 | 46 | 34.6 |
| | Breast conserving surgery (BCS) | 106 | 65.8 | 105 | 66.0 | 87 | 65.4 |
| **Tumour grade at surgery** | 1 – Low | 1 | 0.6 | 1 | 0.6 | 0 | 0 |
| | 2 – Intermediate | 29 | 18 | 29 | 18.2 | 22 | 16.5 |
| | 3 – High | 129 | 80.1 | 127 | 79.9 | 109 | 82.0 |
| | Unobtainable | 2 | 1.2 | 2 | 1.3 | 2 | 1.5 |
| **Tumour size at surgery (mm)** | Median (IQR) | 24 | (15–38) | 24 | (17–36) | 25 | (18–36) |
| **Chemotherapy received** | Neoadjuvant only | 64 | 39.8 | 63 | 39.6 | 49 | 36.8 |
| | Adjuvant only | 46 | 28.6 | 46 | 28.9 | 42 | 31.6 |
| | Neoadjuvant and adjuvant capecitabine | 41 | 25.2 | 40 | 25.2 | 34 | 25.6 |
| | Neoadjuvant and other adjuvant chemo | 10 | 6.2 | 10 | 6.3 | 8 | 6.0 |
| ***BRCA* germline mutation result** | Total with BRCA result | N=101 | | N=100 | | N=99 | |
| | No known mutation | 95 | 94.1 | 94 | 94.0 | 93 | 93.9 |
| | BRCA1 mutation | 3 | 3.0 | 3 | 3.0 | 3 | 3.0 |
| | BRCA2 mutation | 3 | 3.0 | 3 | 3.0 | 3 | 3.0 |

eTable 3: Table demonstrating the clinical characteristics of patients on surveillance in c-TRAK TN.

© 2026 Cunningham N, et al. JAMA Oncol.

| | | Detected by Tissue-Free and Multivariant Tumor-Informed | | Discordant Tissue-Free Detection | | Discordant Multivariant Tumor-Informed Detection | | Not Detected by either Assay | |
|---|---|---|---|---|---|---|---|---|---|
| | | n=41 | | n=4 | | n=6 | | n=82 | |
| | | n | % | n | % | n | % | n | % |
| **Type of definitive surgery** | Mastectomy | 16 | 39.0 | 1 | 25 | 5 | 83.3 | 23 | 28 |
| | Breast conserving surgery (BCS) | 23 | 56.1 | 3 | 75 | 1 | 16.7 | 55 | 67.1 |
| | Other | 2 | 4.9 | 0 | 0 | 0 | 0 | 4 | 4.9 |
| **Tumour grade at surgery** | 1 – Low | 0 | 0 | 0 | 0 | 0 | 0 | 0 | 0 |
| | 2 – Intermediate | 6 | 14.6 | 1 | 25.0 | 2 | 33.3 | 13 | 15.9 |
| | 3 – High | 35 | 85.4 | 3 | 75.0 | 4 | 66.7 | 67 | 81.7 |
| | Unobtainable | 0 | 0 | 0 | 0 | 0 | 0 | 2 | 2.4 |
| **Tumour size at surgery (mm)** | Median (IQR) | 21 | 17-36 | 27 | 24.8-28 | 41.5 | 32.8-56.2 | 24 | 18-34.2 |
| **Clinical Risk Stratification** | High Risk | 23 | 56.1 | 0 | 0 | 4 | 66.7 | 16 | 19.5 |
| | Moderate Risk | 18 | 43.9 | 4 | 100 | 2 | 33.3 | 66 | 80.5 |
| **Recurrence** | Yes | 35 | 85.4 | 0 | 0 | 5 | 83.3 | 4 | 4.9 |
| | No | 6 | 14.6 | 4 | 100 | 1 | 16.7 | 78 | 95.1 |
| **Time to first tissue-free detection from starting surveillance (days)** | Median (IQR) | 104 | 0-248 | 402 | 191-616 | NA | NA | NA | NA |

eTable 4: Table of characteristics of concordant and discordant detection groups in the comparison between tissue-free and tumor-informed assays.

© 2026 Cunningham N, et al. JAMA Oncol.
