# Supplementary material for: Tissue-Free vs Tumor-Informed ctDNA Assays for Molecular Residual Disease Detection in Early Triple Negative Breast Cancer
Source: JAMA Oncol. 2026 Aug 13;12(10):1098–106. doi: 10.1001/jamaoncol.2026.2833 (PMC13474100; doi:10.1001/jamaoncol.2026.2833)
Supplement: Supplementary file 2 [file jamaoncol-e262833-s002.pdf]

Data Sharing Statement

Cunningham. Tissue-Free vs Tumor-Informed ctDNA Assays for Molecular Residual Disease Detection in Early Triple Negative Breast Cancer. *JAMA Oncol*. Published August 13, 2026. doi:10.1001/jamaoncol.2026.2833

Data
Data available: Yes
Data types: Data dictionary, Deidentified participant data
How to access data: De-identified individual participant data, together with a data dictionary defining each field in the set, will be made available to other researchers on request. Trial documentation including the protocol is available on request by contacting c-trak-tn-icrctsu@icr.ac.uk. The ICR-CTSU supports the wider dissemination of information from the research it conducts, and increased cooperation between investigators. Trial data are collected, managed, stored, shared, and archived according to ICR-CTSU Standard Operating Procedures to ensure the enduring quality, integrity, and utility of the data. Formal requests for data sharing are considered in line with ICR-CTSU procedures, with due regard given to funder and sponsor guidelines. Requests are via a standard proforma describing the nature of the proposed research and extent of data requirements. Data recipients are required to enter a formal data sharing agreement, which describes the conditions for release and requirements for data transfer, storage, archiving, publication, and Intellectual Property. Requests are reviewed by the Trial Management Group (TMG) in terms of scientific merit and ethical considerations including patient consent. Data sharing is undertaken if proposed projects have a sound scientific or patient benefit rationale, as agreed by the TMG and approved by the Independent Data Monitoring and Steering Committee, as required. Restrictions relating to patient confidentiality and consent will be limited by aggregating and anonymizing identifiable patient data. Additionally, all indirect identifiers that may lead to deductive disclosures will be removed in line with Cancer Research UK Data Sharing Guidelines.
When available: With publication

Supporting Documents
Document types: None

Additional Information
Who can access the data: N/A
Types of analyses: N/A
Mechanisms of data availability: N/A
